# Esrrb Is a Pivotal Target of the Gsk3/Tcf3 Axis Regulating Embryonic Stem Cell Self-Renewal

**DOI:** 10.1016/j.stem.2012.06.008

**Published:** 2012-10-05

**Authors:** Graziano Martello, Toshimi Sugimoto, Evangelia Diamanti, Anagha Joshi, Rebecca Hannah, Satoshi Ohtsuka, Berthold Göttgens, Hitoshi Niwa, Austin Smith

**Affiliations:** 1 Wellcome Trust - Medical Research Council Stem Cell Institute, University of Cambridge, Cambridge UK; 2 Department of Biochemistry, University of Cambridge, Cambridge UK; 3 Cambridge Institute for Medical Research and Department of Haematology, University of Cambridge, Cambridge UK; 4 RIKEN Center for Developmental Biology, Kobe 650-0047, Japan; 5 Laboratory for Development and Regenerative Medicine, Kobe University Graduate School of Medicine, 7-5-1 Kusunokicho, Chuo-ku, Kobe, Hyogo 6500017, Japan

## Abstract

Inhibition of glycogen synthase kinase-3 (Gsk3) supports mouse embryonic stem cells (ESCs) by modulating Tcf3, but the critical targets downstream of Tcf3 are unclear. We analyzed the intersection between genome localization and transcriptome data sets to identify genes repressed by Tcf3. Among these, manipulations of Esrrb gave distinctive phenotypes in functional assays. Knockdown and knockout eliminated response to Gsk3 inhibition, causing extinction of pluripotency markers and loss of colony forming capability. Conversely, forced expression phenocopied Gsk3 inhibition or Tcf3 deletion by suppressing differentiation and sustaining self-renewal. Thus the nuclear receptor Esrrb is necessary and sufficient to mediate self-renewal downstream of Gsk3 inhibition. Leukaemia inhibitory factor (LIF) regulates ESCs through Stat3, independently of Gsk3 inhibition. Consistent with parallel operation, ESCs in LIF accommodated Esrrb deletion and remained pluripotent. These findings highlight a key role for Esrrb in regulating the naive pluripotent state and illustrate compensation among the core pluripotency factors.

## Highlights

► *Esrrb* is the principal target of Tcf3 repression in the pluripotency network ► Esrrb is essential for self-renewal downstream of Gsk3 inhibition ► Esrrb potently suppresses differentiation and sustains ESC self-renewal ► Esrrb is a core pluripotency factor but can be compensated by LIF/Stat3

## Teaser

Inhibition of glycogen synthase kinase 3 keeps embryonic stem cells in a naïve ground state. Surprisingly this effect is mediated mainly by upregulating expression of a single transcription factor Esrrb. Esrrb function can be compensated by independent activation of Stat3, however, demonstrating the plasticity of the core network underpinning pluripotency.

## Introduction

Since the original derivation of mouse embryonic stem cells (ESCs) in 1981 (Evans and Kaufman, 1981; Martin, 1981), culture conditions for sustaining pluripotency ex vivo have been progressively refined. Following the demonstration that the cytokine leukemia inhibitory factor (LIF) could replace feeder cells (Smith et al., 1988; Williams et al., 1988) and that ESC differentiation is suppressed by inhibition of mitogen activated protein kinase (Erk) signaling (Burdon et al., 1999; Kunath et al., 2007), further addition of an inhibitor of glycogen synthase kinase-3 (Gsk3) has enabled robust ESC propagation in well-defined conditions (Ying et al., 2008). When cultured using the two inhibitors (2i), ESCs display rather uniform marker expression (Wray et al., 2010) and exhibit distinctive gene expression and epigenetic features (Marks et al., 2012). A practical consequence is that it has become facile to establish ESCs from different strains of mice and also rats (Blair et al., 2011). It is noteworthy that, while the triple combination of 2i/LIF appears optimal, mouse ESCs can be propagated by providing any two of these three components (Wray et al., 2011; Wray et al., 2010), implying complementary inputs to a flexible gene regulatory circuit. However, understanding how intracellular signaling pathways engage with the core transcription factor circuitry to maintain or extinguish pluripotency remains fragmentary (Chen et al., 2008; Jaenisch and Young, 2008; Nichols and Smith, 2012; Niwa et al., 2009).

Gsk3 is a negative regulator of many different proteins (Doble and Woodgett, 2003), including transcription factors such as cMyc (Singh and Dalton, 2009). Nonetheless, the effect of Gsk3 inhibition ESC self-renewal is mediated primarily via β-catenin because ESCs lacking β-catenin do not respond productively to Gsk3 inhibitors (Lyashenko et al., 2011; Wray et al., 2011). Consistent with action through intracellular β-catenin, mutation of *Apc* or expression of stabilized β-catenin variants can reduce ESC differentiation (Kielman et al., 2002; Sato et al., 2004). Furthermore, Wnt3a can partially substitute for Gsk3 inhibition and support ESC propagation in conjunction with LIF (ten Berge et al., 2011; Hao et al., 2006; Ogawa et al., 2006; Yi et al., 2011).

It has been suggested that β-catenin might interact directly with Oct4 to promote pluripotent gene expression (Kelly et al., 2011). On the other hand, genetic evidence is incontrovertible that a definitive β-catenin partner, Tcf3 (also known as Tcf7l1), is a major negative regulator of ESC self-renewal (Guo et al., 2011; Pereira et al., 2006). Indeed, ablation of Tcf3 phenocopies deletion or inhibition of Gsk3 (Wray et al., 2011). Genome location analyses indicate that Tcf3 binds in proximity to many core pluripotency genes (Cole et al., 2008; Marson et al., 2008; Tam et al., 2008). Although it has been proposed that β-catenin is recruited to stimulate transcription at these sites (Cole et al., 2008), this model seems inconsistent with the *Tcf3* loss of function phenotype. Furthermore, available evidence indicates that Tcf3 functions in a repressor complex (Pereira et al., 2006; Sokol, 2011; Yi et al., 2011), activity of which can be abrogated by β-catenin without requirement for its transactivation domain (Wray et al., 2011).

Tcf3 has previously been proposed to act via repression of *Nanog* (Pereira et al., 2006). However, *Nanog* null ESCs retain responsiveness to Gsk3 inhibition (Silva et al., 2009), and knockdown of *Nanog* in *Tcf3* null ESCs does not prevent upregulated expression of other pluripotency genes (Yi et al., 2011). It is therefore unclear whether Tcf3 functions as a general repressor of pluripotency genes or acts via a selective target or subset of targets. To resolve this issue, we integrated available high resolution genome location data with transcriptome profiles from Tcf3 perturbation studies. This enabled identification of a small number of transcription factor candidates that were subjected to loss and gain of function analyses.

## Results

### Genome-Scale Identification of Candidate Genes Directly Regulated by Tcf3

**Figure 1:**
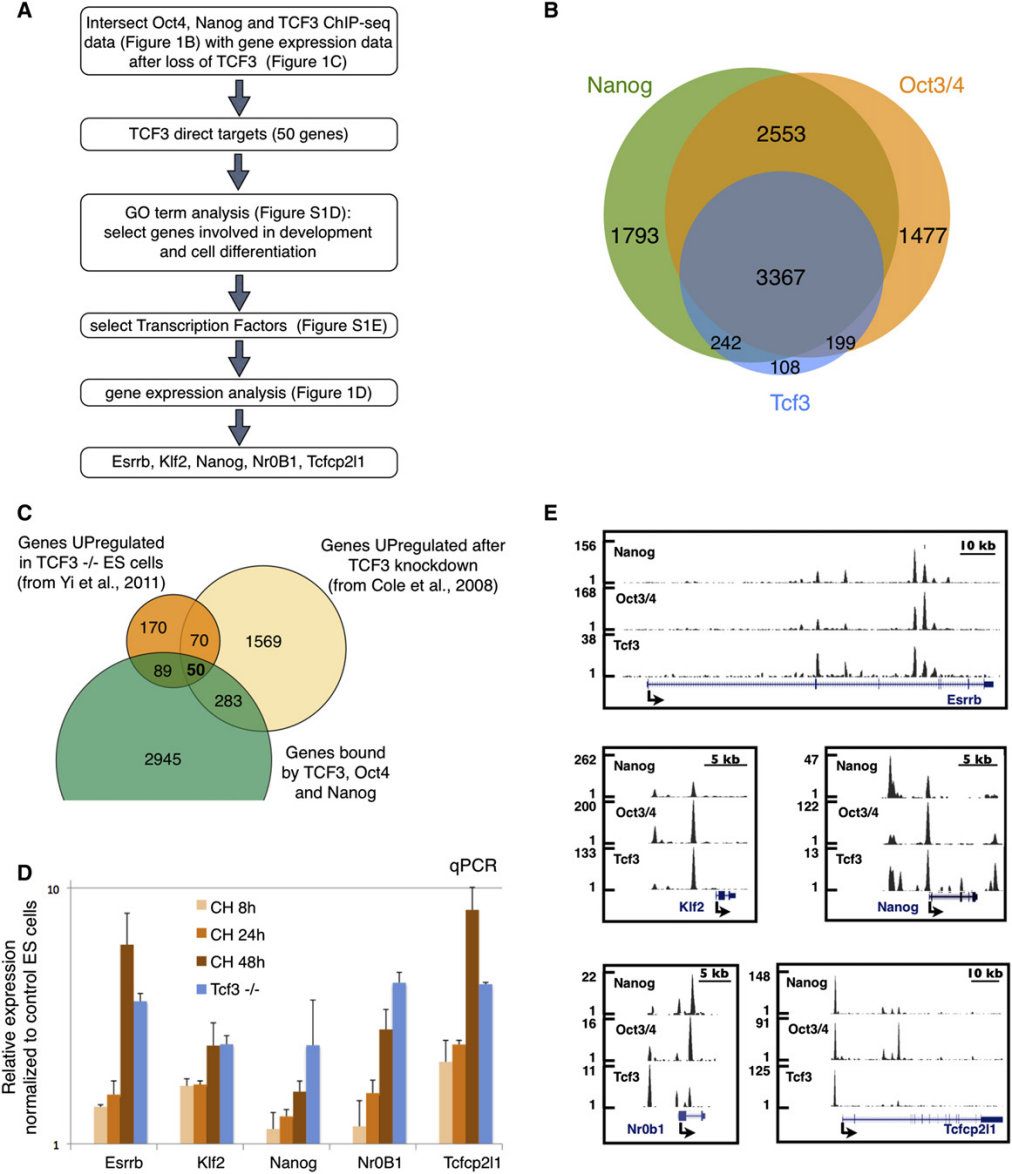
Identification of Tcf3 Direct Targets in Mouse ESCs (A) Flow chart illustrating the approach used to identify candidate genes that mediate self-renewal downstream of Tcf3. (B) Venn diagram showing overlap of Nanog, Oct3/4, and Tcf3 bound genes (see Experimental Procedures). (C) Venn diagram showing overlap between genes upregulated (>1.4-fold change) in Tcf3 null cells, genes upregulated in Tcf3 knockdown, and genes bound by Tcf3, Oct3/4, and Nanog. Fifty genes are identified as candidate Tcf3 direct targets. (D) Gene expression analysis of WT ESCs treated with the Gsk3 inhibitor (CH) for the indicated times (orange columns), and of *Tcf3* null ESCs (blue columns). All cultures were in LIF+serum. The fold change expression relative to WT ESCs treated with vehicle (DMSO) is shown on a logarithmic scale. Mean and SD of two independent experiments is shown. (E) Gene tracks represent binding of Nanog, Oct3/4, and Tcf3 at the indicated gene loci. The x axis represents the linear sequence of genomic DNA and the y axis represents the total number of mapped reads (see Experimental Procedures). See also Figure S1.

Genome-wide mapping of transcription factor binding events is achievable by chromatin immunoprecipitation and deep sequencing (ChIP-seq). Integrated analysis of multiple transcription factor ChIP-seq studies (Hannah et al., 2011), has the potential to provide new mechanistic insights (Ouyang et al., 2009; Wilson et al., 2010). Given the dominant role of transcriptional regulation in ESC function (Jaenisch and Young, 2008; Nichols and Smith, 2012; Niwa, 2007; Smith, 2009; Young, 2011) we generated a compendium of transcription factor ChIP-seq analyses (see Figures S1A and S1B and Table S1 available online and http://bioinformatics.cscr.cam.ac.uk/ES_Cell_ChIP-seq_compendium.html). We used the ChIP-seq compendium to explore candidate target genes for the Tcf3 transcriptional repressor. We focused on those Tcf3 ChIP-seq targets also bound by other pluripotency factors. As previously indicated from promoter-based ChIP-on-chip analysis (Cole et al., 2008), extensive colocalization of Tcf3 with core pluripotency transcription factors Oct4 and Nanog was evident in the genome-wide ChIP-seq data sets (Figure 1B).

We then analyzed published gene expression profiles for ESCs in which *Tcf3* has been inactivated, either by gene targeting (Yi et al., 2011) or by RNAi (Cole et al., 2008). *Tcf3* null and *Tcf3* knockdown ESCs show similarly enhanced self-renewal so we reasoned that genes differentially expressed in both studies are likely to underlie this phenotype. Tcf3 appears to function in ESCs primarily, if not exclusively, as a transcriptional repressor (Pereira et al., 2006; Wray et al., 2011; Yi et al., 2011). We therefore focused on genes with increased expression after *Tcf3* inactivation and found 379 genes upregulated in *Tcf3* null cells and 1,972 in *Tcf3* knockdown cells (Figure 1C). Of these, 120 genes were common to both groups and therefore were considered the most likely specific responders.

We intersected the list of Tcf3 target genes from the ChIP-seq compendium with the 120 upregulated genes and found a statistically significant enrichment (p value = 0.0034), with 50 genes identified as candidates for direct regulation, being both bound by Tcf3 and derepressed after *Tcf3* ablation. The same analysis for genes downregulated following *Tcf3* inactivation did not show significant enrichment for Tcf3 binding (p value = 0.57, Figure S1C), indicating indirect regulation. To survey possible biological roles of Tcf3 direct targets, we performed Gene Ontology analysis and found an overrepresentation of developmental processes such as cell differentiation, anatomical structure development, cell morphogenesis, and embryonic development (Figure S1D). This is in line with the role of Tcf3 as a repressor of pluripotency (Wray et al., 2011) and mediator of axial patterning (Merrill et al., 2004).

**Figure 2:**
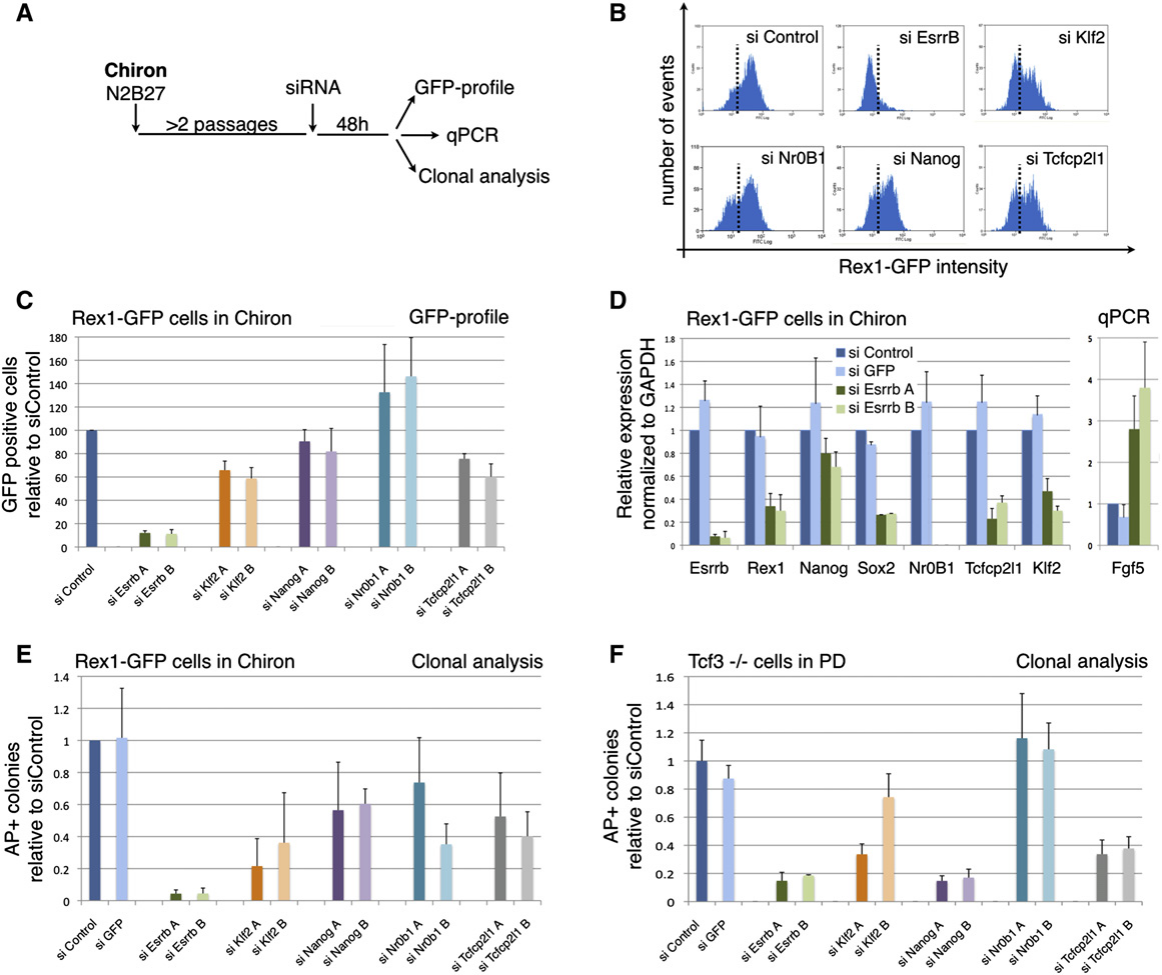
Esrrb Is Required to Mediate Self-Renewal Downstream of GSK3 (A) Experimental scheme for testing the functional requirement of the candidate genes identified in Figure 2. Rex1-GFPd2 cells were cultured in presence of the Gsk3 inhibitor (Chiron-N2B27) for two passages and transfected with two independent siRNAs for each candidate gene. Cells were harvested 48 hr after transfection and analyzed by flow cytometry (Rex1-GFP profile), quantitative PCR, and colony formation. (B) Rex1-GFPd2 cells were transfected with the indicated siRNAs and analyzed after 48 hr by flow cytometry. One representative plot for knockdown of each gene is shown. The dashed line indicates the threshold used to separate GFP-positive and GFP-negative cells. (C) Quantification of the flow cytometry data for Rex1-GFPd2 cells transfected with the indicated siRNAs. Columns show the number of GFP positive cells normalized to the negative control siRNA (siControl). Mean and SD of six independent experiments is shown. (D) Gene expression analysis of Rex1-GFPd2 cells transfected with two independent siRNAs targeting Esrrb or two negative control siRNAs (siControl and siGFP). GAPDH was used as endogenous control and data are normalized to the siControl sample. Mean and SD of four independent experiments is shown. (E) Clonogenicity assay on Rex1-GFPd2 cells transfected with the indicated siRNAs. Forty-eight hr after transfection, cells were replated at clonal density in 2i + LIF and stained for AP after 5 days. Columns show the number of AP+ve colonies normalized to the siControl. Mean and SD of four independent experiments is shown. (F) Clonogenicity assay of *Tcf3* null cells transfected with the indicated siRNAs. Tcf3 null cells cultured in PD were transfected with the indicated siRNAs; after 48 hr they were replated at clonal density in PD and stained for alkaline phosphatase after 5 days. Columns show the number of AP+ve colonies normalized to the negative control siRNA (siControl). Mean and SD of three independent experiments is shown. See also Figures S2B–S2D.

For functional validation, we focused on genes associated with cell differentiation and development and among them selected transcription factors. This gave a short list of nine genes (in bold in Figure S1E). We used gene expression analysis to test responsiveness to the Gsk3 inhibitor Chiron99021 (CH). Four genes showed very low expression with or without CH (data not shown). The other five transcription factor genes exhibited significantly increased expression in response to CH (Figure 2D, orange columns). *Esrrb*, *Klf2*, *Nanog*, *Nr0b1*, and *Tcfcp2l1* thus emerge as candidate mediators of Gsk3/Tcf3-mediated ESC self-renewal. Figure 2E shows gene tracks of Tcf3, Nanog, and Oct3/4 binding at these five gene loci.

We also analyzed *Tcf3* null cells and found an increased level of expression of the five genes, similar to that in wild-type (WT) cells treated with CH (Figure 2D, blue columns). Importantly, when *Tcf3* null cells were treated with CH, we did not observe any further increase in expression (Figure S1F). These data indicate that CH promotes the transcription of *Esrrb*, *Klf2*, *Nanog*, *Nr0b1*, and *Tcfcp2l1* through Tcf3 inhibition without requirement for canonical activation through other Tcf/Lef family members (Yi et al., 2011).

### Esrrb Is Critical for Self-Renewal Downstream of the Gsk3/Tcf3 Axis

Gsk3 deletion or inhibition is sufficient to maintain ESC self-renewal in bulk culture for several passages, albeit with ongoing differentiation (Doble et al., 2007; Wray et al., 2010; Ying et al., 2008). This condition provides a stringent assay for downstream gene requirement. To obtain a quantitative readout, we used Rex1GFPd2 cells, in which a destabilized GFP protein is expressed from the *Rex1* (*Zfp42*) locus (Wray et al., 2011). Rex1 is expressed specifically in ground state pluripotent cells and is downregulated within 24 hr at the onset of differentiation (Toyooka et al., 2008; Wray et al., 2010). Consequently the fraction of undifferentiated ESCs in a population can be quantified by flow cytometric analysis for GFP (Wray et al., 2011). We knocked down the five transcription factor genes in Rex1GFPd2 cells by small interfering RNA (siRNA) transfection (Figure 2A). Two different siRNAs were used for each gene (Figure S2A). Cells were maintained in the presence of CH and evaluated by flow cytometry (Figure 2B). ESCs treated with control siRNA are mostly Rex1GFP-positive with a small shoulder of negative cells. This profile is unaltered by siRNA against *Nr0b1* while *Nanog* knockdown induces only a small increase in the negative fraction. The effect of *Klf2* and *Tcfcp2l1* siRNAs is rather more pronounced, but in both cases the majority of cells remain GFP-positive. In contrast, knockdown of *Esrrb* caused a dramatic shift from Rex1GFP-positive to -negative (Figures 2B and 2C). This is not a selective elimination effect because viable cell numbers determined by live cell gating were similar in all conditions. To exclude nonspecific effects of siRNAs on GFP expression, we confirmed a reduction in Rex1 transcript (Figure 2D). Further qPCR analyses showed a marked downregulation in other pluripotency markers and a concomitant increase in the early differentiation marker Fgf5 upon treatment with siEsrrb.

To address whether decreased expression of Rex1 and pluripotency genes following *Esrrb* knockdown is associated with compromised self-renewal, we tested ability to form undifferentiated colonies 48 hr after siRNA transfection. Cells were plated at single cell density in 2i+LIF and after 5 days stained for alkaline phosphatase (AP). AP activity is a classical marker of undifferentiated ESCs but does not reliably discriminate them from postimplantation epiblast stem cells (EpiSCs) (Bao et al., 2009). However, EpiSCs differentiate or die in 2i (Guo et al., 2009), therefore in this condition the marker is reliably indicative of ESC status. Cells transfected with a control siRNA (siControl) or siRNA targeting *eGFP* (siGFP) gave rise to numerous AP-positive colonies, while knockdown of *Nr0b1*, *Nanog*, *Klf2*, or *Tcfcp2l1* caused up to 2-fold reduction (Figure 2E). Knockdown of *Esrrb* had a much stronger effect, however, almost entirely eliminating AP-positive colonies (Figure 2E, green columns).

Previously, we reported that CH promotes self-renewal by relieving Tcf3 repression (Wray et al., 2011). The expression of each of the five candidate genes is elevated in *Tcf3* null cells (Figure 1D). We therefore examined their importance for the ability of ESCs lacking Tcf3 to self-renew efficiently without requirement for CH. We knocked down the genes in *Tcf3* null cells and tested ability to form colonies in the presence of the Mek inhibitor PD0325901 (PD) alone. Null cells transfected with either siControl or siGFP readily generated AP-positive colonies (Figure 2F, blue columns). Klf2 and Tcfcp2l1 siRNA treatment resulted in slightly fewer colonies, whereas knockdown of Esrrb or Nanog almost eliminated colony formation (Figure 2F, green and purple columns). We also used titrated siRNA to partially reduce *Esrrb* expression to levels close to those in WT ESCs (Figures S2B and S2C). This resulted in restoration of colony formation to similar numbers as for WT ESCs (Figure S2D). Taken together, these results suggest that Esrrb is necessary to mediate self-renewal downstream of Gsk3 inhibition and Tcf3 derepression.

### Esrrb Expression Reproduces the Effect of Gsk3 Inhibition on Self-Renewal

**Figure 3:**
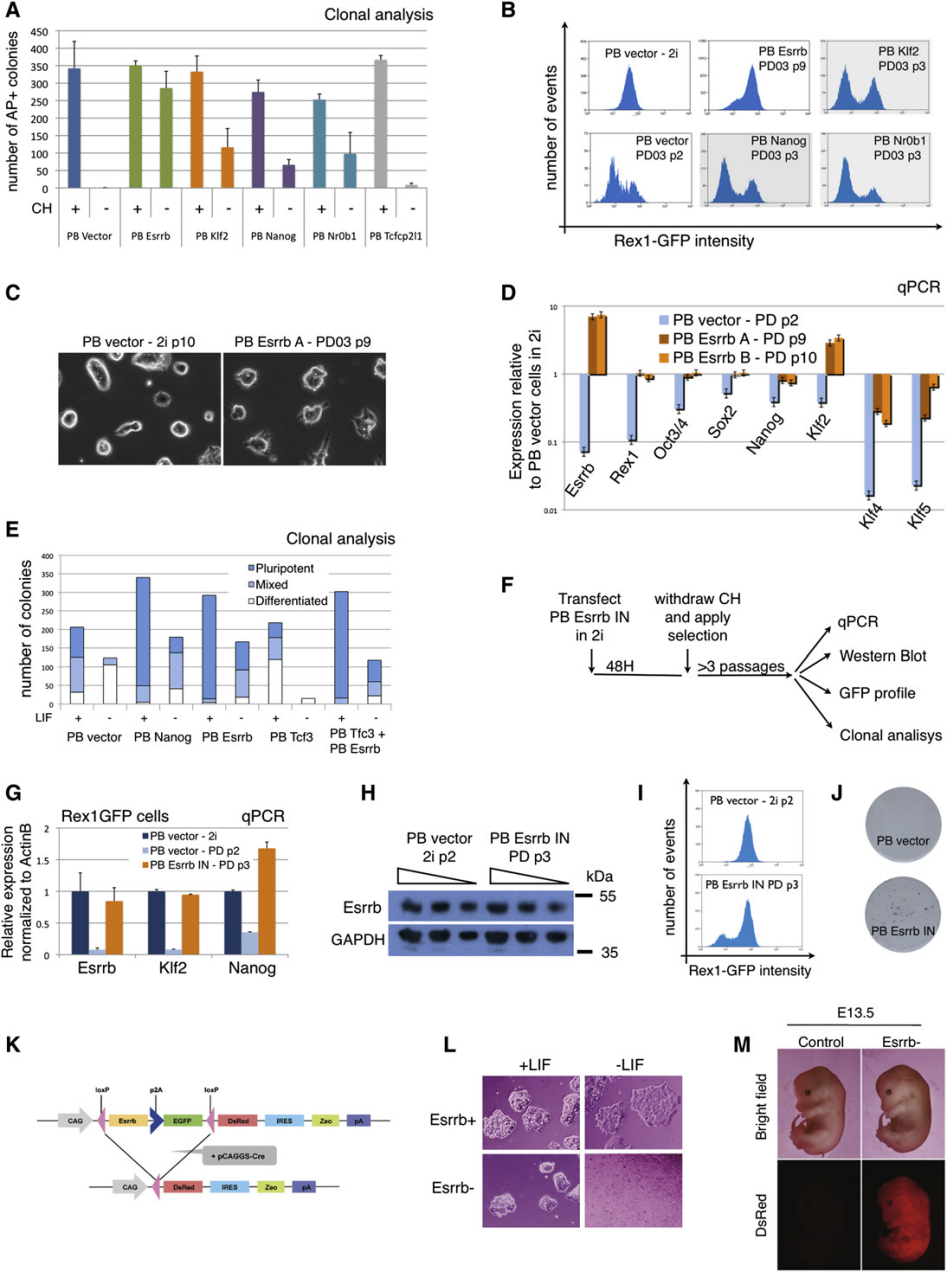
Esrrb Recapitulates the Effect of GSK3 Inhibition on ESC Self-Renewal (A) Rex1-GFPd2 cells were cotransfected with pBase helper plasmid and a piggyBac vector containing *Esrrb*, *Klf2*, *Nanog*, *Nr0b1*, *Tcfcp2l1*, or no cDNA (PB-vector); transfected cells were selected for two passages with Hygromycin in 2i. Six hundred cells were then plated at clonal density in the basal media N2B27 with (+) or without (−) Chiron and stained for AP after 5 days. Columns show the number of AP+ve colonies. Mean and SD of three independent experiments is shown. (B) Flow cytometry analysis of Rex1-GFPd2 transfectants cultured in the indicated conditions. PB-vector cells could not be maintained for more than two passages in presence of the Mek inhibitor PD without CH, whereas PB-Klf2, PB-Nanog, and PB-Nr0b1 cells could not be maintained for more than four passages. Only PB-Esrrb cells showed robust self-renewal under these conditions, expanding continuously for more than 12 passages. (C) Phase contrast pictures of PB-vector transfected cells in 2i media and PB-Esrrb cells in presence of the Mek inhibitor PD. (D) Gene expression analysis of Rex1-GFPd2 cells transfected with either an empty vector or PB-Esrrb and cultured in the indicated conditions. Two independent PB-Esrrb transfections were carried out, generating two independent cell lines named “Rex1-GFPd2 PB-Esrrb A” and “Rex1GFPd2 PB-Esrrb B.” GAPDH was used as endogenous control and data are normalized to PB-vector cells cultured in 2i media. (E) Clonogenicity assay of Rex1-GFPd2 cells transfected with the indicated plasmids and selected for two passages. Six hundred cells were plated at clonal density in Serum media alone or with LIF and stained for AP after 5 days. Columns show the number of wholly AP+ve, mixed or wholly differentiated (AP-ve) colonies. Mean and SD of three independent experiments is shown. See also Figure S3A. (F) Rex1-GFPd2 cells in 2i were cotransfected with pBase helper plasmid and a piggyBac vector containing Esrrb-IRES-Neo (PB Esrrb IN); after 48 hr of culture in 2i, selection was applied and CH was withdrawn. Selection in low G418 (200 mg/ml) allowed isolation of transfectants expressing the *Esrrb* transgene at a level similar to endogenous expression in CH-treated cells. Cells were cultured for >3 passages and analyzed as indicated. (G) Gene expression analysis of Rex1GFP cells transfected with an empty vector (PB-vector) cultured in 2i, in PD for two passages, or Rex1GFP Esrrb IN cells cultured in PD for three passages. Note that in Esrrb IN cells cultured in PD (orange columns), the expression of Esrrb is maintained at levels comparable to control cells cultured in 2i (blue columns). ActinB served as an internal control. (H) Western blot of Rex1GFP cells transfected with an empty vector (PB-vector) cultured in 2i, and Rex1GFP Esrrb IN cells cultured in PD for three passages. For each sample, three different amounts of total proteins (equivalent to 50,000, 25,000, and 12,500 cells) were loaded. Note that Esrrb protein levels are comparable in the two samples. GAPDH served as a loading control. (I) Rex1GFP cells transfected with an empty vector (PB-vector) cultured in 2i for three passages, and Rex1GFP Esrrb IN cells cultured in PD for three passages have been analyzed by flow cytometry. Seventy-nine percent of Esrrb IN cells were GFP-positive. (J) Rex1GFP cells transfected with an empty vector (PB-vector) cultured in 2i for three passages, and Rex1GFP Esrrb IN cells cultured in PD for three passages have been plated at clonal density in N2B27+PD and stained for alkaline phosphatase after 5 days. As expected, PB-vector cells could not form AP+ colonies (top panel), whereas Rex1GFP Esrrb IN cells did (bottom panel). (K) Cre excisable construct used for *Esrrb* overexpression. (L) Representative pictures of cells transfected with the Esrrb excisable construct before (Esrrb+) and after excision (Esrrb-). Note that only Esrrb+ cells can be cultured in serum containing media without LIF. Successful excision was confirmed by PCR using transgene-specific primers (Figure S3H). (M) *Esrrb* excised ESCs contribute to chimeric embryos. The DsRed signal is specifically detected in the injected embryos at midgestation and not in a sibling embryo with no chimeric contribution (control).

We then examined whether expression of *Esrrb* or the other factors may be sufficient to substitute for Gsk3 inhibition. Rex1GFPd2 cells maintained in 2i were transfected with Piggybac expression vectors (Guo et al., 2009). Following hygromycin selection for two passages in 2i, the response to inhibitor withdrawal was tested in colony forming assays and in bulk culture (Figures 3A and 3B). PB-vector and PB-Tcfcp2l1 transfectants yielded negligible colonies when plated at clonal density in N2B27 alone. PB-Nr0b1, PB-Nanog, and PB-Klf2 produced some colonies but many fewer than in the presence of CH. PB-Esrrb, however, generated undifferentiated colonies in comparable number and size to those obtained in the presence of CH. The absence of any significant additive effect when Esrrb is overexpressed in the presence of CH (Figure 3A) suggests that Esrrb is capable of fully recapitulating the effect of Gsk3 inhibition on ESC clonogenicity.

In bulk culture in the presence of PD alone, PB-vector and PB-Tcfcp2l1 cells collapsed within two passages. Other transfectants could be maintained longer but progressively lost Rex1GFP expression (Figure 3B) and completely differentiated or died by five passages. By contrast, PB-*Esrrb* cells could easily be expanded in PD for more than 12 passages. Their Rex1GFP profile showed only a small shoulder of GFP low cells and the cells retained undifferentiated morphology (Figures 3B and 3C). PB-Esrrb cells in these conditions expressed *Oct4*, *Sox2*, and *Nanog* at a level similar to control cells in 2i (Figure 3D, orange columns). They showed reduced *Klf4* and *Klf5* but increased *Klf2*.

We then tested whether expression of *Esrrb* could confer clonal self-renewal in the presence of serum, which is normally dependent on LIF. Colonies were scored as undifferentiated, mixed, or differentiated according to the AP staining pattern because serum supports the survival and proliferation of differentiated cells. PB-vector cells formed undifferentiated colonies only in the presence of LIF, whereas PB-Esrrb cells were equally capable of generating AP-positive colonies without LIF (Figure 3E), similarly to PB-Nanog cells that serve as a positive control. Strikingly, the combination of *Esrrb* overexpression and LIF caused a further increase in the number of undifferentiated colonies, indicating that Esrrb is in a parallel pathway to LIF/Stat3. We also used this assay to examine whether Esrrb could rescue the differentiation phenotype induced by overexpression of Tcf3. Indeed the generation of undifferentiated colonies is diminished by transfection with PB-Tcf3 alone and regained by cotransfection with PB-Esrrb (Figure 3E). Consistent with the colony assay, Tcf3 transfectants showed reduced expresson of *Nanog* that was restored by Esrrb (Figure S3A).

Esrrb-expressing cells formed undifferentiated AP-positive colonies when LIF or Wnt signaling were blocked by a Jak inhibitor or DKK respectively (Figures S3B and S3C). Furthermore, neither the LIF target *Socs3* nor the Wnt target *Axin2* showed elevated expression (Figures S3D and S3E), confirming that Esrrb does not act by stimulating these pathways. We also cocultured GFP labeled Esrrb transfectants with unlabelled WT cells. After three passages in serum without LIF, the entire population was GFP-positive (Figure S3F), demonstrating that Esrrb acts cell autonomously.

In unscreened PB-Esrrb cells, the expression of Esrrb is typically up to 6- to 8-fold higher than endogenous levels. This creates the possibility of neomorphic effects. We therefore transfected Rex1GFPd2 ESCs with an alternative PB-Esrrb vector allowing capture of low expressors using G418 (Figure 3F). We screened for level of Esrrb expression and identified a pool in which Esrrb messenger RNA (mRNA) and protein are constitutively expressed at levels comparable to those found in control vector transfectants in 2i (Figures 3G and 3H). These PB-Esrrb-neo cells recapitulate the phenotype of Esrrb overexpressing cells: in the absence of CH or LIF, they can be cultured for multiple passages, they express pluripotency markers (Figures 3G and 3I), and they self-renew at clonal density (Figure 3J). We conclude that constitutive expression of *Esrrb* at endogenous levels is sufficient to sustain ESC self-renewal.

Because constitutive expression of Esrrb blocks differentiation, transgene excision (Chambers et al., 2003; Niwa et al., 2009) was necessary to determine whether ESCs maintained by Esrrb retain pluripotency. After transfection with an excisable vector, cells were clonally selected and expanded in serum without LIF for 1 month. They were then transiently transfected with a Cre expression vector followed by subcloning of DsRed-positive cells in the presence of LIF (Figure 3K). These subclones no longer expressed the *Esrrb* transgene and reacquired dependency on LIF (bottom panels of Figure 3L and Figure S3H). Reverted cells were injected into blastocysts and gave rise to chimeric embryos in which DsRed-expressing cells contributed widely (Figure 3M).

Collectively, these results indicate that constitutive expression of *Esrrb* can replace Gsk3 inhibition and furthermore maintain self-renewal and pluripotency independently of LIF/Stat3.

### Esrrb Is Dispensable for Self-Renewal in the Presence of LIF

**Figure 4:**
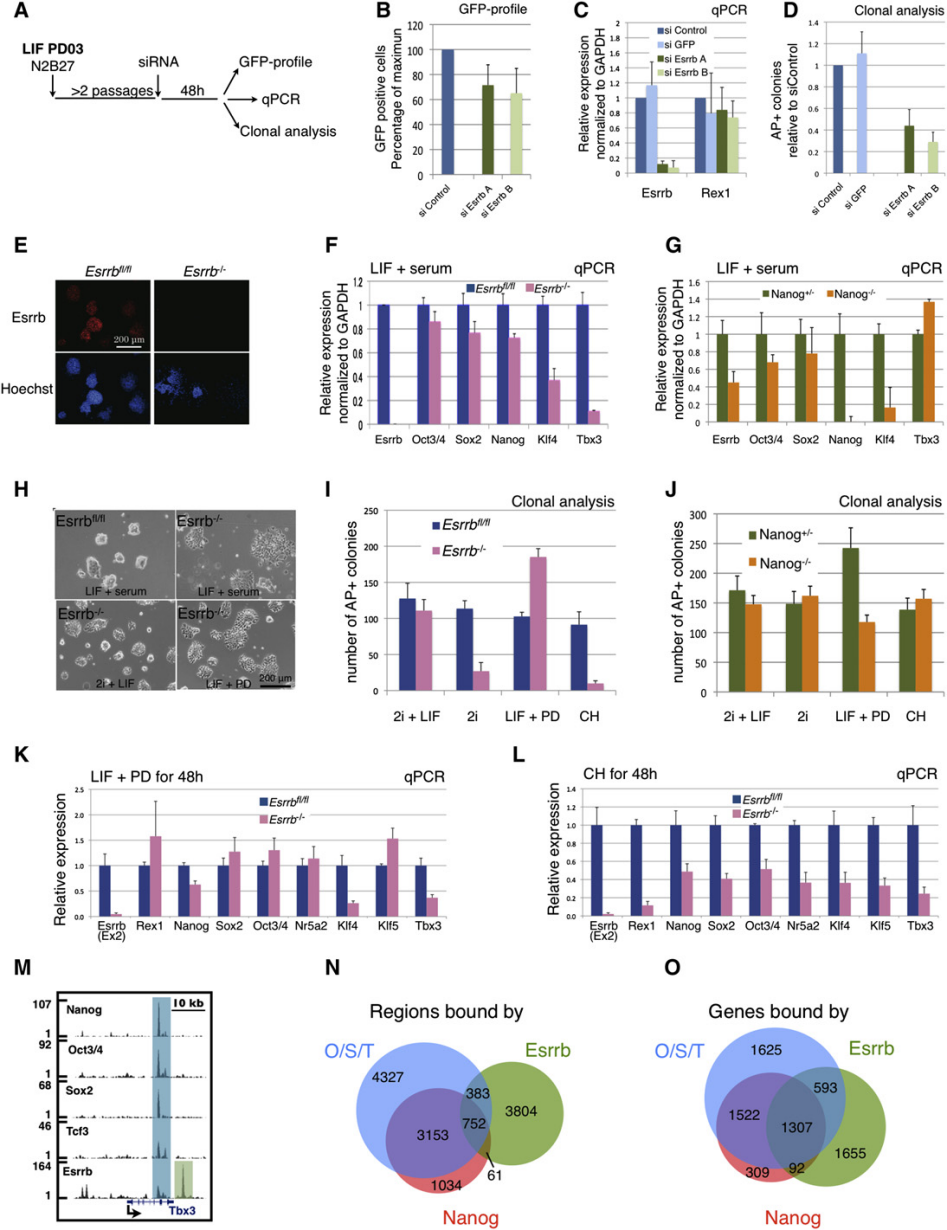
Esrrb Is Not Required for Self-Renewal in the Presence of LIF (A) Rex1-GFPd2 cells were cultured in N2B27 plus LIF and PD03 for two passages and transfected with two independent siRNAs targeting Esrrb or two negative control siRNAs (siControl and siGFP). Cells were harvested 48 hr after transfection and analyzed by flow cytometry (Rex1-GFP profile), quantitative PCR, and clonal analysis. (B) Rex1-GFPd2 cells were transfected with the indicated siRNAs and analyzed after 48 hr by flow cytometry. Columns show the number of GFP-positive cells normalized to the negative control siRNA (siControl). Mean and SD of three independent experiments is shown. (C) Gene expression analysis of Rex1-GFPd2 cells transfected with two independent siRNAs targeting Esrrb or two control siRNAs (siControl and siGFP). GAPDH was used as endogenous control and data are normalized to the siControl. Mean and SD of three independent experiments is shown. (D) Quantification of clonogenicity assay of Rex1-GFPd2 cells transfected with the indicated siRNAs. Forty-eight hr after transfection, cells were replated at clonal density in 2i media and stained for AP after 5 days. Columns show the number of AP+ve colonies normalized to the siControl. Mean and SD of three independent experiments is shown. (E) Immunostaining of *Esrrb*^−/−^ and *Esrrb*^fl/fl^ cells confirming absence of Esrrb protein in the *Esrrb*^−/−^ cells. (F) Gene expression analysis of *Esrrb*^−/−^ and *Esrrb*^fl/fl^ cells cultured in LIF+Serum. Note the absence of Esrrb transcript. GAPDH was used as endogenous control and data are normalized to *Esrrb*^fl/fl^ cells. (G) Gene expression analysis of *Nanog*^−/−^ and *Nanog*^+/−^ cells cultured in LIF+Serum. Note the absence of Nanog transcript. GAPDH was used as endogenous control and data are normalized to *Nanog*^+/−^ cells. (H) Phase contrast images of *Esrrb*^fl/fl^ and *Esrrb*^−/−^ cells under the indicated culture conditions. (I and J) Colony forming assay on *Esrrb*^fl/fl^ and *Esrrb*^−/−^ (I), and *Nanog*^−/−^ and *Nanog*^+/−^ (J) cells. Cells were cultured in 2i+LIF, plated at clonal density (300 cells per well) under the indicated conditions, and stained for alkaline phosphatase after 5 days. Columns show the number of AP+ve colonies. Mean and SD of three independent experiments is shown. (K and L) *Esrrb*^fl/fl^ and *Esrrb*^−/−^ ESCs were cultured for 48 hr in LIF+PD (K) or CH (L) and analyzed by qPCR for the indicated pluripotency markers. GAPDH was used as endogenous control and data are normalized to Esrrb^fl/fl^. Mean and SD of two independent experiments is shown. (M) Gene tracks representing binding of Nanog, Oct3/4, Sox2, Tcf3, and Esrrb at the Tbx3 gene locus. The x axis represents the linear sequence of genomic DNA, and the y axis represents the total number of mapped reads. A blue box highlights a region where the five factors colocalize while the green box highlights a region bound only by Esrrb. (N) Venn diagram showing the intersection between the genomic regions bound by at least one factor among Oct3/4, Sox2, and Tcf3 (O/S/T in blue), by Nanog (in red) and by Esrrb (in green). For this diagram, only the top 5,000 ChIP-Seq peaks of each factor have been used, in order to account for differences in ChIP efficiency among different factors. See also Figure S4C. (O) Venn diagram showing the intersection between the predicted target genes bound by at least one factor among Oct3/4, Sox2, and Tcf3 (O/S/T in blue), and the predicted target genes of Nanog (in red) or Esrrb (in green). Only the top 5,000 peaks for each factor have been used to predict target genes in order to account for differences in ChIP efficiency. See also Figure S4D and Table S5.

Although Esrrb appears essential for ESC propagation downstream of Gsk3 inhibition, the forced expression studies indicate that it does not lie downstream of LIF/Stat3. Therefore *Esrrb* might be dispensable in the presence of LIF, just as *Stat3* can be deleted when Gsk3 is inhibited (Ying et al., 2008). We tested this hypothesis by knocking down *Esrrb* in Rex1GFPd2 cells cultured in the presence of LIF and PD (Figure 4A). Under these conditions we found that *Esrrb* siRNA had a modest effect on the naive pluripotency marker Rex1 (Figures 4B and 4C) and caused only a partial reduction in the ability to form undifferentiated colonies (Figure 4D). These findings are in stark contrast with the near elimination of self-renewal by Esrrb siRNA in the absence of LIF (Figure 2).

To substantiate these findings, we generated ESCs in which the *Esrrb* gene can be conditionally inactivated. Through serial gene targeting, we inserted loxP sites to flank the second exon of both alleles of *Esrrb* (Figure S4A). The second exon encodes the start codon and part of the DNA-binding domain. *Esrrb*^−/−^ ESCs generated by Cre-mediated recombination are therefore expected to be functionally null. Tamoxifen-regulatable Cre was used to effect deletion. Homozygous deletion was confirmed in a clonally expanded population by immunostaining and qPCR (Figures 4E and 4F). These *Esrrb* null ESCs remained morphologically undifferentiated in serum-containing medium with LIF over multiple passages. Expression of some pluripotency-associated genes, *Klf4* and *Tbx3*, was reduced (Figure 4F) and the null cells exhibited a rather more flattened morphology (Figure 4H), but they showed similar proliferation to WT ESCs. Interestingly, *Nanog*^−/−^ ESCs also show reduced expression of *Klf4* (Figure 4G). From examination of the ChIP-seq compendium, *Klf4* is a likely direct target of both Esrrb and Nanog, which may explain its lower level in null cells.

When *Esrrb* null ESCs were cultured in serum-free conditions, they expanded in 2i+LIF and LIF+PD. They were less compact than parental cells but remained undifferentiated (Figure 4H) with no overt compromise of proliferation or viability. However, on LIF withdrawal, the null cells collapsed within one passage. Thus, in the absence of Esrrb, ESCs lose the ability to respond effectively to Gsk3 inhibition and their propagation appears strictly dependent on LIF. Clonal analysis of *Esrrb*^−/−^ cells confirmed these findings, showing a dramatic reduction in the number of AP-positive colonies in the absence of LIF (Figure 4I, compare 2i+LIF to 2i and LIF+PD to CH).

We carried out gene expression analysis after 48 hr of culture either in LIF+PD or in CH (Figures 4K and 4L). In LIF+PD, *Esrrb*^fl/fl^ and *Esrrb*^−/−^ cells showed a comparable profile, apart from reduced *Klf4* and *Tbx3* in the null cells (Figure 4K). In contrast, after 48 hr in CH *Esrrb*^−/−^ cells showed lower expression of all pluripotency markers (Figure 4L). These deletion findings are fully consistent with results obtained after *Esrrb* knockdown (Figures 2 and 4A–4D), confirming that Esrrb is essential for self-renewal downstream of CH, but can be compensated for by LIF stimulation. In contrast, *Nanog* null cells could be propagated under all conditions described above, consistent with previous observations (Silva et al., 2009) that Nanog is not required for responsiveness to Gsk3 inhibition (Figure 4J).

To explore why loss of Esrrb has more severe consequences than deletion of Nanog, we utilized the ChIP-seq compendium (Figure S1) to compare profiles of Nanog, Oct4, Sox2, Tcf3, and Esrrb. As shown in Figure 4M using the *Tbx3* locus as an example, there are regions bound by Nanog, Oct4, Sox2, Tcf3 (O/S/T) together with Esrrb (see blue box), as well as regions bound only by Esrrb (see green box). Global analysis revealed that a majority of the genomic regions bound by Nanog are co-occupied by at least one of Oct4, Sox2, and Tcf3 (O/S/T), as well as Esrrb. However, only a minority of Esrrb-bound regions are occupied by any of the other four pluripotency factors (Figure 4N; Figure S4C). To investigate whether Esrrb-specific occupancy translates into Esrrb-specific candidate target genes, we mapped binding peaks to genes (see Table S1 and Experimental Procedures section) and intersected the resulting gene lists. Whereas over 90% (2,921 out of 3,230) of the predicted Nanog target genes were also targets of O/S/T and/or Esrrb, only ∼55% (1,992 out of 3,647) of Esrrb candidate targets were shared with any of the other factors (Figure 4O; Figure S4D). The extensive overlap of Nanog with Oct4, Sox2, Tcf3, and/or Esrrb candidate targets may explain why Nanog deletion can be tolerated in established ESCs. Conversely, the wider occupancy of Esrrb is consistent with the observation that Esrrb function is less readily dispensable.

**Figure 5:**
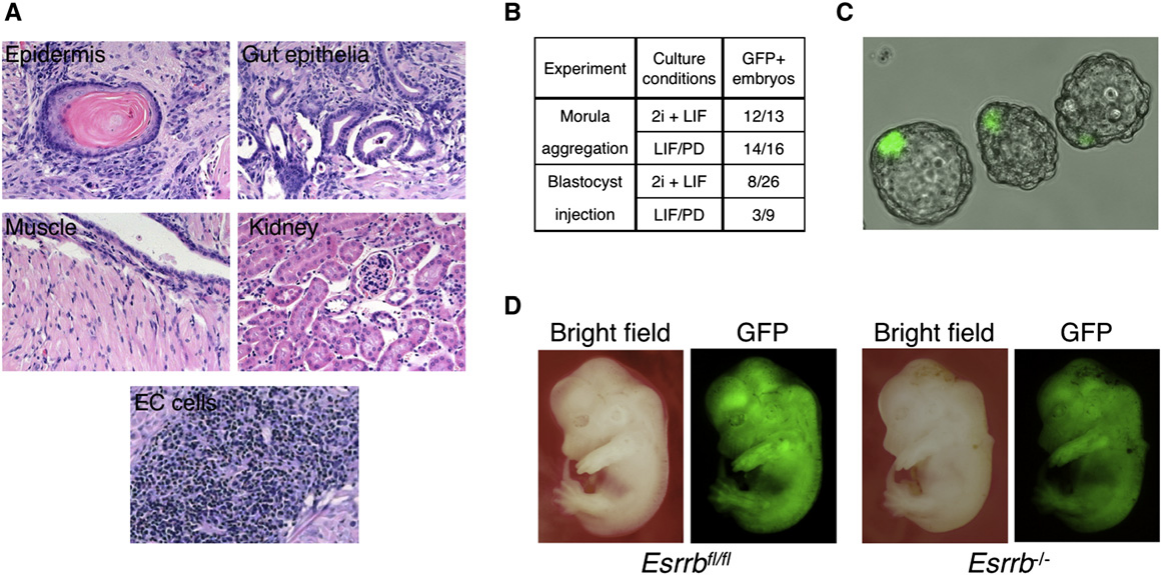
Esrrb Null Cells Are Pluripotent (A) *Esrrb*^−/−^ ESCs cultured in LIF+serum were injected into the kidney capsule and produced teratocarcinomas containing tissues representative of the three germ layers (epidermis for ectoderm, kidney and striated muscle for mesoderm and gut-like epithelia for endoderm) along with undifferentiated embryonal carcinoma (EC) cells. (B) *Esrrb*^−/−^ ESCs contribute to chimeric embryos: summary of the experiments performed with GFP-labeled *Esrrb*^−/−^ ESCs. See also Figures S5A and S5B. (C) GFP-labeled *Esrrb*^−/−^ ESCs cultured in either 2i+LIF or LIF/PD, were combined with 8-cell stage embryos, cultured in vitro for 48 hr, and scored for the presence of GFP-positive cells in the ICM. A representative image is shown. (D) Blastocyst injection was performed followed by embryo transfer and embryos were scored at midgestation (E12.5) for the presence of GFP-positive cells. Esrrb fl/fl served as a positive control. See also Figures S5A and S5B.

We then examined whether ESCs lacking Esrrb and maintained using LIF are pluripotent. Cells grafted under the kidney capsule gave rise within 6 weeks to large multidifferentiated tumors that contained neural, mesodermal, and endodermal tissues along with undifferentiated embryonal carcinoma (Figure 5A). The tumors showed no overt differences in size or differentiation from teratocarcinomas generated by *Esrrb*^fl/fl^ cells. Most significantly, the embryonic identity and developmental potential of *Esrrb* null cells was confirmed by integration into the inner cell mass after aggregation with morulae (Figures 5B and 5C) and widespread contribution to the midgestation embryo after blastocyst injection (Figure 5D; Figure S5).

### Direct Regulation of Esrrb Expression by the Gsk3/β-Catenin/Tcf3 Axis

**Figure 6:**
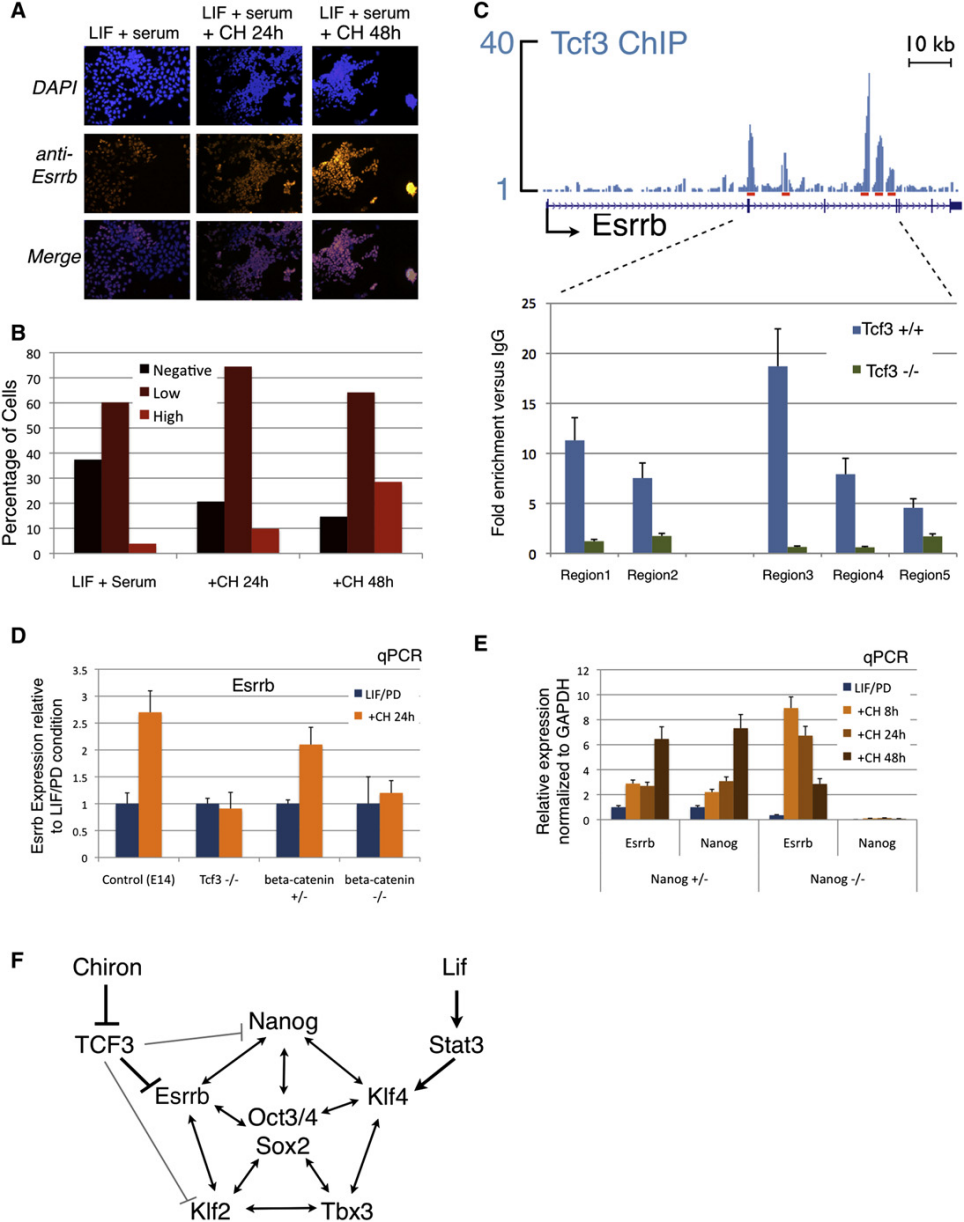
Mechanism of Esrrb Regulation by Tcf3 (A) Fluorescence micrographs showing immunostaining for Esrrb of WT ESCs cultured under the indicated conditions. (B) Histogram showing the distribution of Esrrb immunostaining intensity under the indicated conditions. More than 3,000 single ESCs for each condition were analyzed and divided into three categories based on staining intensity (see Experimental Procedures section). (C) Top shows that gene tracks represent binding of Tcf3 at the *Esrrb* gene locus. The five red boxes indicate the regions analyzed by ChIP for Tcf3. Bottom shows that ChIP for Tcf3 followed by qPCR for the indicated regions was performed in either WT or Tcf3 null cells. Enrichment over a mock ChIP is shown. Mean and SD of three independent experiments is shown. (D) Esrrb expression analysis of the indicated ESC lines, cultured in LIF/PD and treated with the Gsk3 inhibitor (CH) for 24 hr (orange columns). The fold change expression relative to LIF/PD is shown; ActinB served as an internal control. Mean and SD of two independent experiments is shown. (E) Gene expression analysis of *Nanog* +/− and −/− cells, cultured in LIF/PD and treated with the Gsk3 inhibitor (CH) for 8 hr or 24 hr (orange columns). GAPDH served as an internal control. Mean and SD of two independent experiments is shown. (F) Schematic representation of core pluripotency transcription factor circuit with parallel input from LIF/Stat3 and GSK3 inhibition/Tcf3 derepression. See also Figure S6.

In keeping with several other core pluripotency factors, Esrrb is expressed in a mosaic fashion in ESCs cultured in serum. When CH is added to the cultures, however, Esrrb immunostaining becomes more uniform and increased in intensity (Figures 6A and 6B). To confirm binding of Tcf3 to the *Esrrb* gene, we carried out single point ChIP assays at five regions detected in previous ChIP studies (Cole et al., 2008; Marson et al., 2008). Marked enrichment was observed in WT ESCs compared with *Tcf3* null cells (Figure 6C). As noted above, *Tcf3* null ESCs show elevated expression of Esrrb that is not further induced by CH alone. We repeated this analysis in the presence of LIF and PD and obtained similar results (Figure 6D). *β-catenin* null ESCs also fail to upregulate Esrrb in response to CH, consistent with their inability to self-renew without LIF (Wray et al., 2011). These data confirm that both Tcf3 and β-catenin are necessary for *Esrrb* induction by Gsk3 inhibition. We found that Wnt3a can induce *Esrrb*, although to lower levels than CH (Figure S6), likely reflecting the operation of negative feedback in the canonical Wnt pathway that can be short-circuited by Gsk3 inhibition. Finally, we investigated the possibility that induction of Esrrb may involve Nanog. Nanog is modestly upregulated by both CH and Tcf3 ablation (Figure 1D) and it has been shown to bind to the *Esrrb* promoter region (Chen et al., 2008). We found that Esrrb is fully induced by CH in *Nanog* null ESCs (Figure 6E). Steady state expression is lower in the absence of Nanog, however (see also Figure 4G). We conclude that Nanog plays a role in maintenance of Esrrb expression level, but that induction by Gsk3 inhibition is mediated directly by abrogation of Tcf3 repression (Figure 6F).

## Discussion

Several mechanisms have been proposed through which inhibition of Gsk3 may stabilize the naive state of mouse ESCs (Cole et al., 2008; Kelly et al., 2011; Sokol, 2011; Wray et al., 2011; Yi et al., 2011). The present analyses clarify this debate by identifying *Esrrb* as a direct functional target of Tcf3 that is derepressed downstream of Gsk3 inhibition. Perturbation studies demonstrate that Esrrb activity is both necessary and sufficient for the response to Gsk3 inhibition. Notably, Esrrb exhibits markedly more potent capacity than other Tcf3 targets to suppress differentiation, sustain propagation, and maintain key pluripotency genes. While recognizing that Tcf3 also regulates other key genes in the pluripotency circuitry, these findings pinpoint Esrrb as the main effector through which the Gsk3/β-catenin/Tcf3 axis modulates ESC self-renewal.

We generated a compendium of publicly available ChIP-seq data for 38 transcriptional regulators in mouse ESCs (Figures S1A and S1B). This integrated resource with a unified data structure enables streamlined cross-referencing of individual data sets from different laboratories. Distinct clusters of binding profiles can be discerned that appear to partition the ESC transcriptional program into several major subcompartments. The compendium thus provides a powerful analytical tool with the potential to fuel new hypotheses on the transcriptional control of ESC function. Tcf3 binds at a large number of sites, including many genes implicated in ESC biology (Cole et al., 2008; Marson et al., 2008; Tam et al., 2008). We used the compendium resource to examine in greater detail genes bound by both Tcf3 and core pluripotency factors and generate a refined list of candidate targets, which included Esrrb.

Esrrb is an orphan nuclear receptor related to the estrogen receptor (Luo et al., 1997). A potential role in ESCs was previously suggested from results of an RNAi screen by Ivanova and colleagues who found that knockdown of *Esrrb* reduced ESC self-renewal efficiency and promoted differentiation (Ivanova et al., 2006). Subsequently, Esrrb was reported to replace Klf4 in fibroblast reprogramming, albeit with reduced efficiency (Feng et al., 2009). Through protein interaction studies, Esrrb was found to bind to Oct4 and cooperate in transcriptional regulation of *Nanog* (van den Berg et al., 2008). Esrrb is also proposed to interact with Nanog and thereby play a reciprocal role in regulation of *Oct4* expression (Zhang et al., 2008). These authors also reported that overexpression of Esrrb can support formation of AP colonies in serum in the absence of LIF (Zhang et al., 2008). However, the identity of the colonies was not characterized further and neither self-renewal nor retention of pluripotency were investigated. Nor is there any evidence that Esrrb is induced by LIF. In the embryo, deletion of *Esrrb* causes midgestation lethality due to placental defects (Luo et al., 1997), but mutant embryos develop through implantation and gastrulation. Furthermore, tetraploid complementation rescued embryo development at least to midgestation (Luo et al., 1997), confirming that there is no defect in the ICM or epiblast. Hence the functional significance of Esrrb in ESCs and its position in the regulatory hierarchy have been uncertain.

Here, through loss-of-function studies by siRNA knockdown and definitive gene deletion via homologous recombination, we established that *Esrrb* is specifically required for the self-renewal effect of Gsk3 inhibition. Furthermore, *Esrrb* gain-of-function can replace Gsk3 inhibition and support long-term ESC propagation in the presence of Mek inhibition alone. Constitutive expression of Esrrb can also confer clonogenic LIF-independent self-renewal in serum. Importantly, ESCs propagated under the direction of Esrrb remain pluripotent and capable of colonizing chimeras when the transgene is removed.

Mouse ESC self-renewal is highly responsive to LIF, acting through Jak kinase and the downstream transcription factor Stat3 (Matsuda et al., 1999; Niwa et al., 1998). Neither LIF nor Gsk3 inhibition alone can fully suppress ESC differentiation, but the combination of both together is very effective (Wray et al., 2010). Furthermore, *Stat3* null ESCs can be derived and maintained using 2i and are functionally nonresponsive to LIF (Ying et al., 2008), whereas β-catenin null ESCs self-renew in the presence of LIF but do not respond to Gsk3 inhibition (Lyashenko et al., 2011; Wray et al., 2011). These observations indicate that Gsk3 inhibition/Tcf3 derepression supports self-renewal in parallel with LIF/Stat3 and inputs independently into the core pluripotency network (Figure 6F). Indeed, although *Esrrb* expression can act dominantly to confer LIF independence, addition of LIF further augments clonogenic capacity. Consistent with parallel pathways, the requirement for Esrrb is not absolute. ESCs lacking *Esrrb* remain undifferentiated if cultured in the presence of LIF with either PD or serum. Thus the need for Esrrb, just as for Gsk3 inhibition or β-catenin (Lyashenko et al., 2011; Wray et al., 2011), is conditional on whether ESCs receive other pro- and antidifferentiation stimuli, in particular LIF. Parallel compensatory capacity could explain why the pluripotent lineage in the early embryo can develop without Esrrb (Luo et al., 1997).

When *Esrrb* knockdown or knockout ESCs are cultured without LIF, they downregulate pluripotency genes and exit self-renewal. We therefore conclude that Esrrb is a component of the core transcription factor network that underpins pluripotency. Esrrb appears to be highly interconnected with other pluripotency factors by both protein interactions and transcriptional cross-regulation (van den Berg et al., 2008; van den Berg et al., 2010; Zhang et al., 2008). Interestingly, examination of the ChIP-seq compendium reveals that although Esrrb binds at many of the same genes as other core pluripotency factors (Nanog, Oct4, Sox2, Tcf3), it often occupies different sites. In addition, Esrrb is located at a large group of genes not bound by the other factors. These observations are consistent with the functional evidence that Esrrb makes a distinctive contribution to ESC self-renewal. Indeed, we find that Esrrb binding is detected at ∼70% of genes (8,149 out of 12,051) that show significant expression by RNA-seq (>0.5 RPKM) in ground state ESCs (Marks et al., 2012). This suggests that it may play a generalized role in ESC transcription, particularly when considered with evidence that Esrrb interacts physically with components of the basal transcriptional machinery (van den Berg et al., 2010).

In summary, these findings place Esrrb on a par with Nanog, Klf4, Klf2, and Tbx3 in the pantheon of intrinsic pluripotency factors that together with Oct4 and Sox2 establish and sustain naive ESCs (Figure 6F). Specifically, Esrrb is a direct target of Tcf3 repression and the principal factor mediating the self-renewal response to Gsk3 inhibition and stabilization of β-catenin. Esrrb acts independently of LIF/Stat3, conferring flexibility and robustness to naive ESC self-renewal. Future work will explore the mutual compensation and plasticity in the pluripotency gene regulatory network that allows LIF stimulation to accommodate deletion of *Esrrb*. It will also be of interest to examine epiblast development in blastocysts doubly deficient for *Esrrb* and *Stat3*. Finally, we note that *Esrrb* is not significantly expressed in mouse postimplantation epiblast stem cells (EpiSCs), nor in currently available human pluripotent stem cells, neither of which show a self-renewal response to Gsk3 inhibition.

## Experimental Procedures

### Generation of ChIP-seq Compendium

Processed data files (density maps, wig format; peak coordinates, bed format) were acquired from the NCBI Gene Expression Omnibus (GEO) and Short Sequence Read Archive (SRA). They were displayed in the UCSC Genome Browser allowing a visual inspection of the ChIP-seq data and peak calling quality. Where the published peak coordinates appeared to over- or underpredict, or were unavailable, creation of density maps and peak calling was performed de novo using the mapped reads as described (Hannah et al., 2011). The specific peak parameters used are indicated in Table S1.

A binary peak matrix was generated as described (Hannah et al., 2011), analyzed by unsupervised hierarchical clustering using Pearson correlation coefficients, and displayed using the heatmap function in R (Figure S1A).

Lists of candidate target genes for all factors have been generated by peak-to-gene mapping using the same uniform parameters for all studies, which may be different from the parameters used in the individual original studies. The requirements used are as follows: if a TF peak is within 100 bp of the TSS, it is associated with that gene alone; all other peaks can be associated with up to two genes, by examining 50 kb of flanking sequence on either side of the peak.

The lists of candidate targets have been analyzed by unsupervised hierarchical clustering using Pearson correlation coefficients, and displayed using the heatmap function in R (Figure S1B). All raw and processed data files used for this study are available for download from http://bioinformatics.cscr.cam.ac.uk/ES_Cell_ChIP-seq_compendium.html. The gene tracks have been generated by loading the density maps of the indicated factors into the UCSC genome browser as custom tracks.

The ChIP-seq data used for peaks intersection, target gene intersection, and gene tracks (in Figures 1 and 4) are as follows: Esrrb, GSE11431 (GSM288355); Nanog, GSE11724 (GSM307140 and GSM307141); Oct4, GSE11724 (GSM307137); Sox2, GSE11724 (GSM307138 and GSM307139); Tcf3, GSE11724 (GSM307142 and GSM307143).

### Embryonic Stem Cell Culture

ESCs were cultured without feeders on plastic coated with 0.1% gelatine (Sigma, cat. G1890) and replated every 3 days at a split ratio of 1 in 10 following dissociation with Accutase (PAA, cat. L11-007). Cells were cultured either in the GMEM (Sigma, cat. G5154) supplemented with 10% FCS (Sigma, cat. F7524), 100 μM 2-mercaptoethanol (Sigma, cat. M7522), 1× MEM nonessential amino acids (Invitrogen, cat. 1140-036), 2 mM L-glutamine, 1 mM sodium pyruvate (both from Invitrogen), and 100 units/ml LIF, or in the serum-free media N2B27 (NDiff N2B27 base medium, Stem Cell Sciences Ltd, cat. SCS-SF- NB-02) supplemented, as indicated, with small-molecule inhibitors PD (1 μM, PD0325901) and CH (3 μM, CHIR99021) and LIF prepared in-house. Colony forming assays were carried out by plating 600 ESCs per well on plates coated with laminin (Sigma, cat. L2020). Plates were fixed and stained for AP (Sigma, cat. 86R-1KT) according to the manufacturer’s protocol. Plates were scanned using a CellCelector (Aviso) and scored manually.

### Gene Expression Analysis by Quantitative PCR with Reverse Transcription

Total RNA was isolated using the RNeasy Kit (QIAGEN) and complementary DNA (cDNA) was made from 500 ng using SuperScriptIII (Invitrogen) and oligo-dT primers. For real-time PCR, we used TaqMan Fast Universal Master Mix and TaqMan probes (Applied Biosystems) or the Universal Probe Library (UPL, Roche) system. In Figures 4F, 4J, and 4K, we used SYBR green system. Primers and UPL probe numbers are detailed in Table S2. Technical replicates were carried out for all quantitative PCR reactions. An endogenous control (Gapdh, glyceraldehyde-3-phosphate dehydrogenase, Applied Biosystems 4352339E) was used to normalize expression.

### RNAi Experiments

siRNAs were transfected at a final concentration of 40 nM using Dharmafect 1 (Dharmacon, cat. T-2001-01), following the protocol for reverse transfection. For a 12-well plate (4cm^2^), we used 2 μl of transfection reagent, 2 μl of 20 microM siRNA solution, and 30,000 ESCs in 1 ml of N2B27 medium. The medium was changed after overnight incubation; 48 hr after transfection, the cells were analyzed as indicated. See Table S3 for sequences of the siRNAs used.

### Flow Cytometry

After treatment with Accutase, live ESCs were resuspended in PBS with 3% FCS and ToPro-3 (Invitrogen) was added at a concentration of 0.05 nM to detect dead cells. Flow cytometry analyses were performed using a Dako Cytomation CyAn ADP high-performance cytometer with Summit software.

### Essrb Gene Targeting

LoxP sites were inserted to flank exon2 of *Esrrb* using a promoter-trap vector containing an FRT flanked SA-IRES-βgeo selection cassette (Figure S4). Subsequent transfection with the FLPe expression vector removed this cassette to generate *Esrrb*^fl/+^ ESCs. Targeting was confirmed by genomic PCR. This strategy was repeated for the second allele to create homozygous *Esrrb*^fl/fl^ ESCs. To enable efficient conditional deletion, we stably transfected cells with an expression vector for the tamoxifen-inducible form of Cre (MerCreMer). Null cells were obtained by treatment with 200ng/ml of Tamoxifen for 3 days and expanded from single cells in medium containing serum and LIF.

Mouse studies were carried out in a designated facility under licenses granted by the UK Home Office.

## Supplemental Information

Document S1. Figures S1–S6, Tables S1–S4, and Supplemental Experimental Procedures
