# Supplementary material for: Esrrb Is a Pivotal Target of the Gsk3/Tcf3 Axis Regulating Embryonic Stem Cell Self-Renewal
Source: Cell Stem Cell. 2012 Oct 5;11(4):491–504. doi: 10.1016/j.stem.2012.06.008 (PMC3465555; doi:10.1016/j.stem.2012.06.008)
Supplement: Document S1. Figures S1–S6, Tables S1–S4, and Supplemental Experimental Procedures [file mmc1.pdf]

Current Biology, Volume *22*

Supplemental Information

Esrrb Is a Pivotal Target of the Gsk3/Tcf3 Axis Regulating Embryonic Stem Cell Self-Renewal

Graziano Martello, Toshimi Sugimoto, Evangelia Diamanti, Anagha Joshi, Rebecca Hannah, Satoshi Ohtsuka, Berthold Göttgens, Hitoshi Niwa, and Austin Smith

Supplemental Inventory

1. Supplemental Figures and Tables

Figure S1, related to Figure 1 in paper

Figure S2, related to Figure 2 in paper

Figure S3, related to Figure 3 in paper

Figure S4, related to Figure 4 in paper

Figure S5, related to Figure 5 in paper

Figure S6, related to Figure 6 in paper

Table S1, related to Figure S1

Tables S2–S4, related to Experimental Procedures

Table S5, see separate Excel document

2. Supplemental Experimental Procedures

A

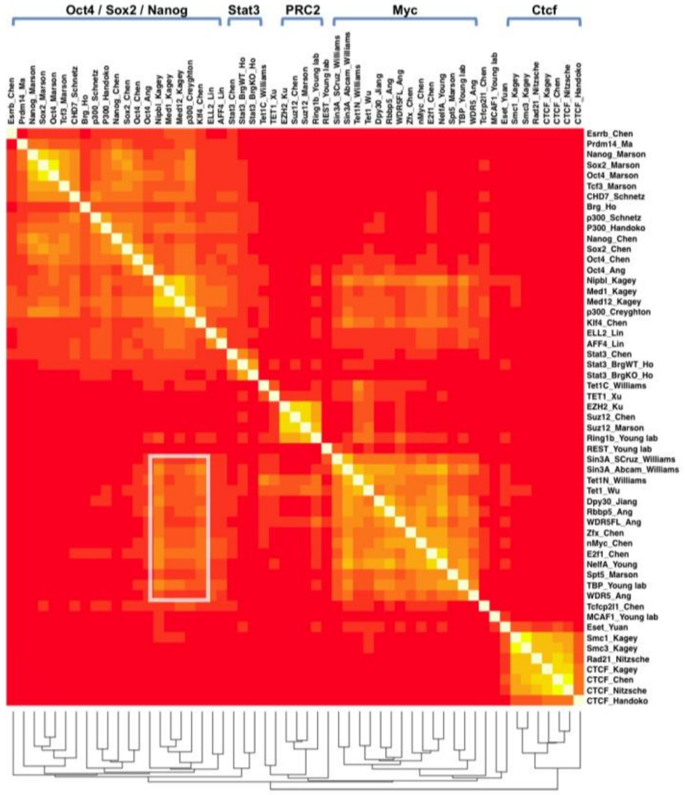

B

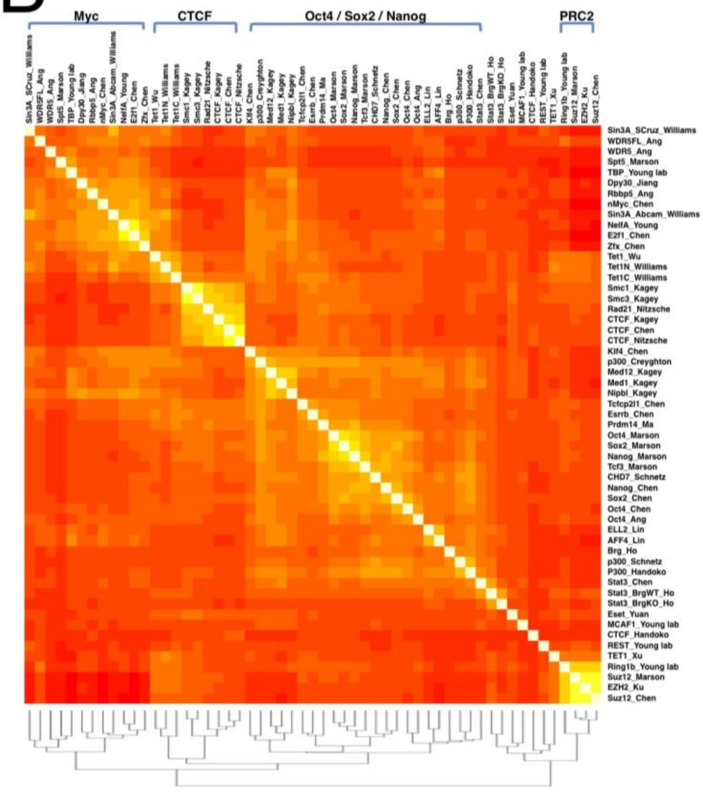

C

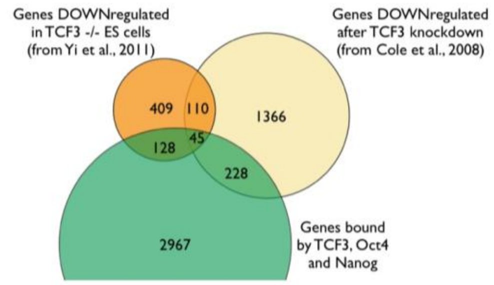

D

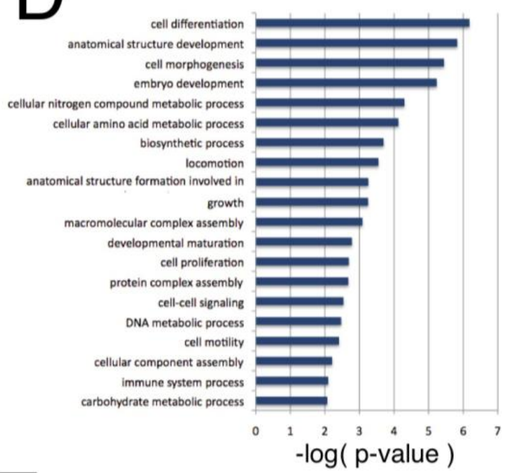

E

Gene (**TF** in bold)	cell differentiation	anat. struct. development	cell morphogenesis
COBL		X	
**ESRRB**	X	X	X
**ETV4**	X	X	X
FUT9		X	
**GLI2**	X	X	X
GRSF1		X	
**KLF15**	X	X	
**KLF2**	X	X	X
LIFR	X	X	X
MAPT	X	X	X
MCF2	X	X	
**MORC1**	X		
**NANOG**	X	X	
**NR0B1**	X	X	
**TCFCP2L1**	X	X	X
TUBB2B	X	X	
VEGFC	X	X	

F

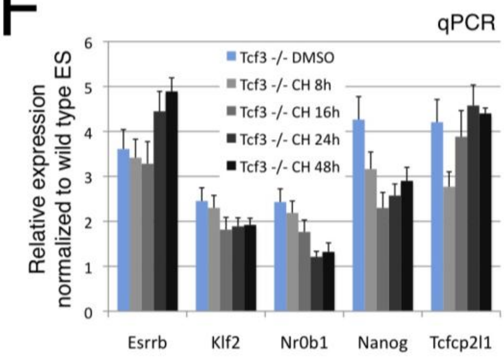

Figure S1. Related to Figure 1

(A) Hierarchical clustering of 55 genome-wide binding maps. For analysis of combinatorial binding patterns, a unified data matrix was generated based on the 165,607 unique peak regions, indicating for each factor whether it was bound or not to each of the 165,607 unique regions. To investigate global relationships, hierarchical clustering and Pearson's correlation coefficients were used to display all pairwise comparisons in a clustered heatmap. Colours in the heatmap show the level of correlation for all pairwise comparisons (white is correlation=1; red is correlation=0). Factors have been clustered along both axes according to the level of correlation. The five major clusters identified are indicated at top of the figure by blue brackets. The first cluster contains "classical" pluripotency genes such as Oct4 and Nanog, together with more general transcriptional regulators such as p300. The second cluster comprises three STAT3 studies, while the third cluster contains polycomb repressor complex 2 (Prc2) components. The fourth is centred around N-Myc and contains NelfA and Spt5, which like Myc are involved in the control of transcriptional pause release (Rahl et al., 2010), plus a subcluster of Trithorax components (Dpy30, Rbpp5 and Wdr5). The final cluster contains CTCF and other factors thought to be involved in chromosome domain structure. Binding maps can show correlation with more than one major cluster, for example the Nipl/Med1/Med12/p300 cluster shows similarities to both the Oct4/Sox2/Nanog cluster and the Myc cluster (see white box).

(B) Hierarchical clustering of 55 factors based on predicted target genes. For each factor a list of candidate target genes has been compiled and used to calculate the correlation for all pairwise comparisons (see Methods section for details). Heatmap analysis generated the same 5 major clusters (compare Figures S1A and S1B). For most transcription factors therefore, the distinct clustering observed in Figure S1A is not attributable to binding different regions within the same gene loci, but instead reflects different gene targets. However, a small number of factors such as Esrrb that did not belong to any major cluster in figure 1A, were placed into the Oct4/Sox2/Nanog cluster in the target-gene based heatmap (Figure S1B). This suggests that such factors control target genes overlapping with the core pluripotency targets, but often do so through distinct binding sites. Data are represented as in Figure S1A.

(C) Venn diagram showing overlap between genes down-regulated in Tcf3 null cells, genes downregulated in Tcf3 knockdown and genes bound by Tcf3, Oct3/4 and Nanog.

(D) Gene ontology (GO) term analysis of up-regulated candidate Tcf3 direct targets revealed a statistically significant enrichment for genes involved in embryonic development and cell differentiation. GO categories are ranked according to the p-value. Similar results were obtained using the 3367 genes bound by Nanog, Oct4 and Tcf3 as the background, thereby explicitly asking whether any GO categories are specifically enriched in the 50 genes when considered as a subset of the 3367 genes (data not shown).

(E) Table containing the Tcf3 targets found in at least 1 of the 3 most represented GO categories. The genes in bold encode transcription factors.

(F) Gene expression analysis of *Tcf3* null ES cells, cultured in LIF+serum, treated either with the Gsk3 inhibitor (CH) for the indicated time (gray bars) or with DMSO (vehicle, blue bars). The fold change expression relative to wt ES cells treated with vehicle (DMSO) is shown. Mean and SD of 2 independent experiments is shown.

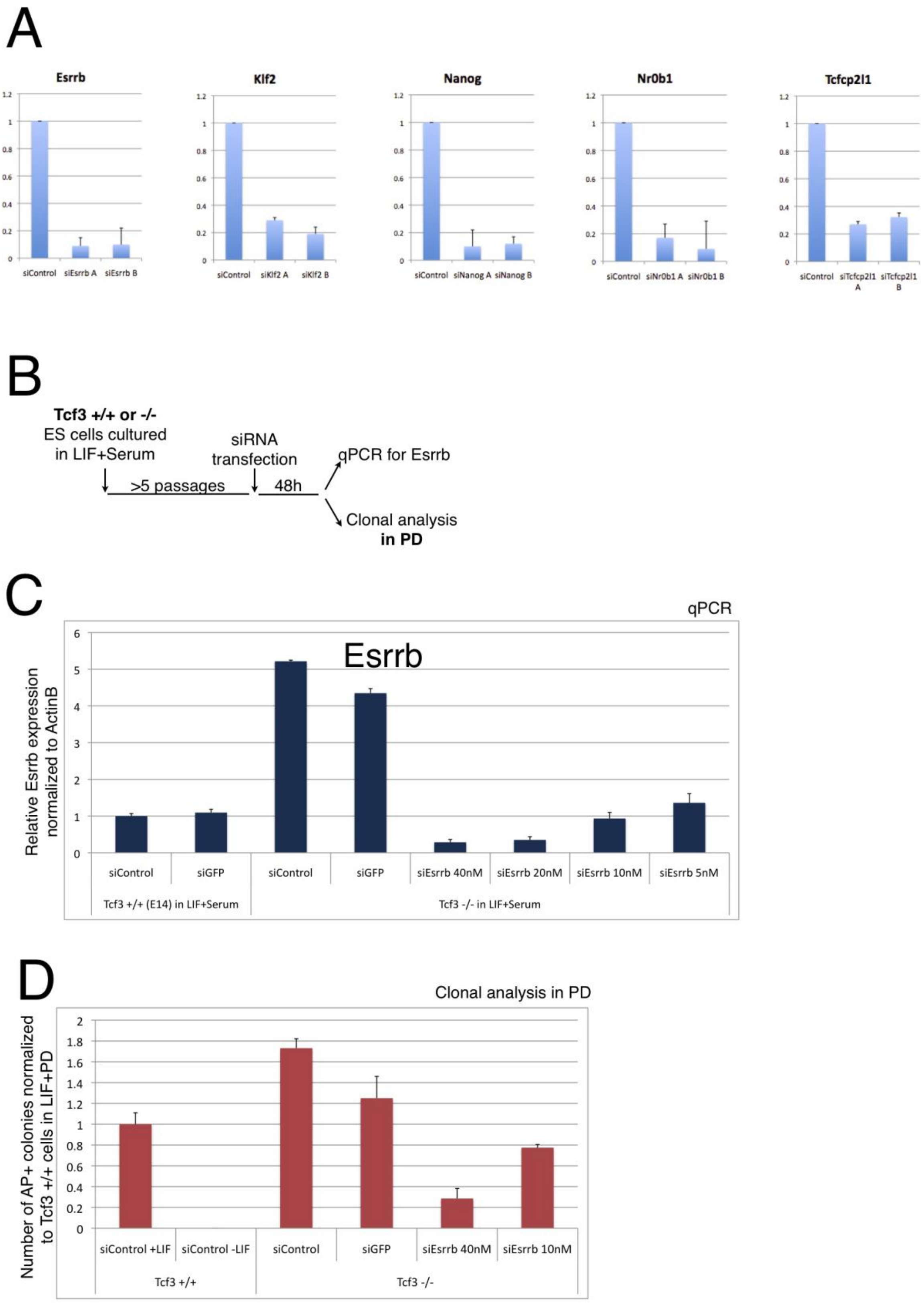
A
Esrrb
siControl siEsrrb A siEsrrb B
Klf2
siControl siKlf2 A siKlf2 B
Nanog
siControl siNanog A siNanog B
Nr0b1
siControl siNr0b1 A siNr0b1 B
Tcfcp2l1
siControl siTcfcp2l1 A siTcfcp2l1 B
B
Tcf3 +/+ or -/-
ES cells cultured
in LIF+Serum
>5 passages
siRNA
transfection
48h
qPCR for Esrrb
Clonal analysis
in PD
C
qPCR
Esrrb
Relative Esrrb expression
normalized to ActinB
siControl
siGFP
siControl
siGFP
siEsrrb 40nM
siEsrrb 20nM
siEsrrb 10nM
siEsrrb 5nM
Tcf3 +/+ (E14) in LIF+Serum
Tcf3 -/- in LIF+Serum
D
Clonal analysis in PD
Number of AP+ colonies normalized
to Tcf3 +/+ cells in LIF+PD
siControl +LIF
siControl -LIF
siControl
siGFP
siEsrrb 40nM
siEsrrb 10nM
Tcf3 +/+
Tcf3 -/-

Figure S2. Related to Figure 2

(A) Validation of the siRNAs used in this study. ES cells were transfected with the indicated siRNAs and analyzed after 24h by qPCR for the 5 indicated genes. For each genes 4 independent siRNAs have been tested and the 2 showing the best knockdown efficiency have been used. Data in the figure are average and standard deviation of 2 independent experiments.

(B) *Tcf3* -/- cells, or Tcf3 +/+ (E14) , were cultured in LIF+serum media and transfected with the indicated siRNAs. After 48h cells were analyzed by qPCR and replated at clonal density in N2B27 media in presence of PD.

(C) Gene expression analysis of Tcf3 -/- and +/+ cells transfected with 2 control siRNAs (siControl and siGFP) or with different concentrations of siEsrrb. As expected, Esrrb expression is elevated in Tcf3 -/- cells, compared to Tcf3 +/+; trasfection of siEsrrb at 10 nM concentration reduced Esrrb espression to levels found in control cells (Tcf3+/+) transfected with negative control siRNAs. ActinB was used as endogenous control and data are normalized to Tcf3 +/+ cells trasfected with the control siRNA (siControl). Mean and SD of 2 independent experiments is shown.

(D) Clonogenicity assay of *Tcf3* -/- and +/+ cells transfected with the indicated siRNAs. Cells cultured in LIF+serum were transfected with the indicated siRNAs at the indicated concentration; after 48h they were replated at clonal density in PD, and stained for alkaline phosphatase after 5 days. Bars show the number of AP+ve colonies normalized to Tcf3 +/+ cells trasfected with the negative control siRNA (siControl) plated in LIF+PD, that also served as a positive control. Note that siEsrrb at 40nM concentration abolished colony formation (see also Figure 2F), while at the 10nM concentration colony formation was restored to levels comparable to Tcf3+/+ cells in LIF+PD. Mean and SD of 2 independent experiments is shown.

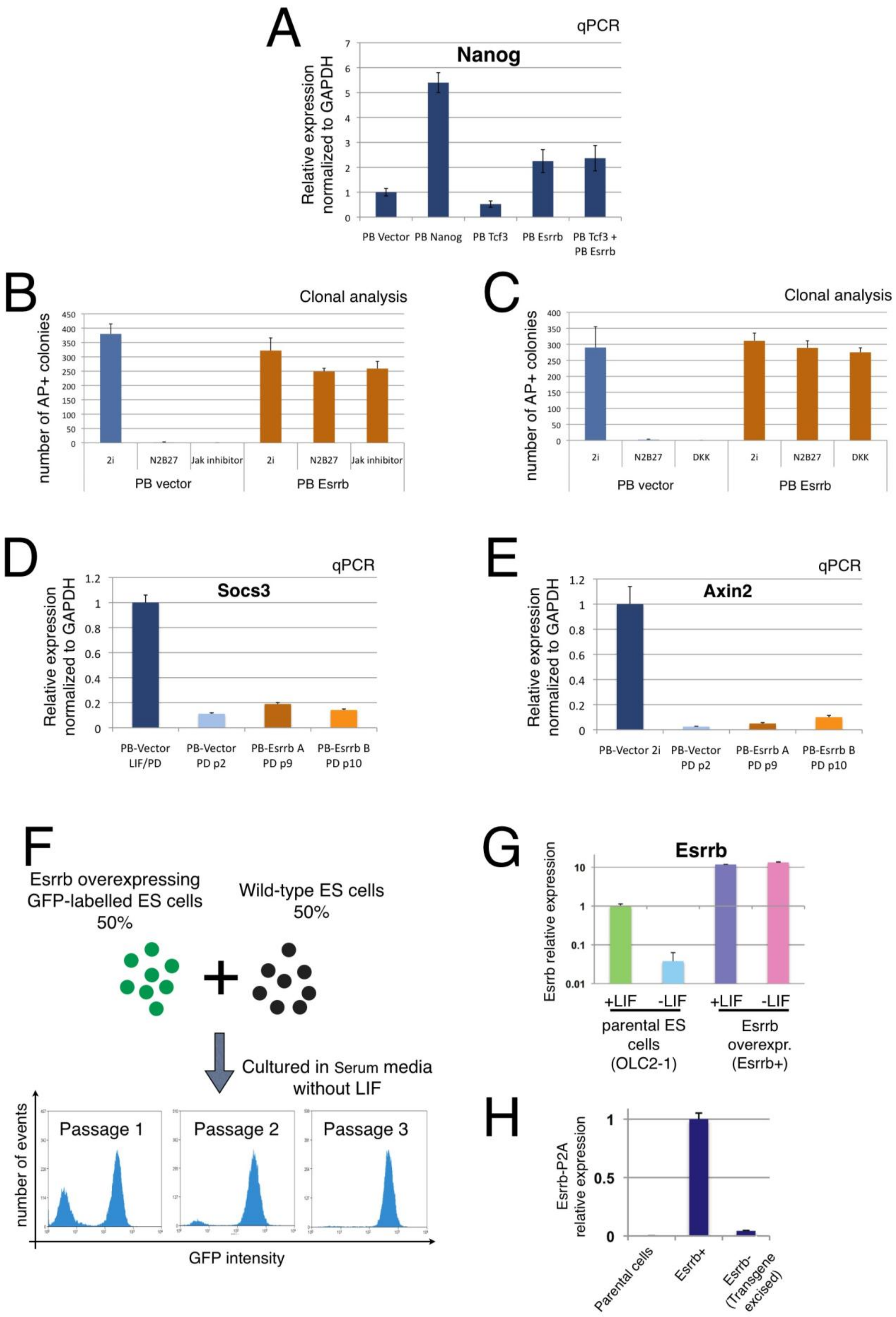

A
qPCR
Nanog
Relative expression normalized to GAPDH
PB Vector
PB Nanog
PB Tcf3
PB Esrrb
PB Tcf3 + PB Esrrb
B
Clonal analysis
number of AP+ colonies
2i
N2B27
Jak inhibitor
PB vector
PB Esrrb
C
Clonal analysis
number of AP+ colonies
2i
N2B27
DKK
PB vector
PB Esrrb
D
qPCR
Socs3
Relative expression normalized to GAPDH
PB-Vector LIF/PD
PB-Vector PD p2
PB-Esrrb A PD p9
PB-Esrrb B PD p10
E
qPCR
Axin2
Relative expression normalized to GAPDH
PB-Vector 2i
PB-Vector PD p2
PB-Esrrb A PD p9
PB-Esrrb B PD p10
F
Esrrb overexpressing GFP-labelled ES cells 50%
Wild-type ES cells 50%
Cultured in Serum media without LIF
Passage 1
Passage 2
Passage 3
number of events
GFP intensity
G
Esrrb
Esrrb relative expression
+LIF
-LIF
+LIF
-LIF
parental ES cells (OLC2-1)
Esrrb overexpr. (Esrrb+)
H
Esrrb-P2A relative expression
Parental cells
Esrrb+
Esrrb- (Transgene excised)

Figure S3. Related to Figure 3

(A) Expression levels of Nanog measured by qPCR in the indicated cell lines. Expression is normalized to empty vector transfected ES cells cultured in LIF and serum. Gapdh was used as internal control. Mean and SD of 2 biological replicates are shown.

(B) Representative pictures (bottom) and quantification (top) of clonogenicity assay on Rex1-GFPd2 cells transfected with either PB-vector or PB-Esrrb and cultured in the indicated conditions. 600 cells were plated at clonal density in 2i, in the basal media N2B27 or in N2B27 + Jak inhibitor (0.6microM), and stained for alkaline phosphatase (AP) after 5 days. Bars show the number of AP+ve colonies. Mean and standard deviation of 3 replicas of 2 independent experiments are shown.

(C) Representative pictures (bottom) and quantification (top) of clonogenicity assay on Rex1-GFPd2 cells transfected with either PB-vector or PB-Esrrb and cultured in the indicated conditions. 600 cells were plated at clonal density in 2i, in the basal media N2B27, or in N2B27 plus the Wnt inhibitor DKK (200ng/ml), and stained for alkaline phosphatase (AP) after 5 days. Bars show the number of AP+ve colonies. Mean and SD of 3 replicates from 2 independent experiments are shown.

(D) Expression levels of the LIF target Socs3. Socs3 levels were measured by qPCR and normalized to parental cells cultured in LIF/PD. Gapdh was used as internal control. Mean and SD of 2 experiments are shown.

(E) Expression levels of the Wnt target Axin2. Axin2 levels were measured by qPCR and normalized to parental cells cultured in 2i. Gapdh was used as internal control. Mean and SD of 2 experiments are shown.

(F) Esrrb sustains self-renewal in a cell autonomous manner. Wild-type ES cells were mixed with GFP-labelled Esrrb overexpressing cells in a 1:1 ratio. The mixed population of cells were cultured in the absence of LIF and the GFP intensity was measured by flow cytometry over 3 passages. Note that the wild-type, GFP negative, cells were rapidly lost indicative of differentiation, confirming that Esrrb overexpressing cells maintain self-renewal in a cell-autonomous manner. Similar results were obtained by culturing the mixed population in N2B27 media in presence of PD (data not shown).

(G) Gene expression analysis of parental and Esrrb overexpressing (Esrrb+) cells cultured in the indicated conditions. Total Esrrb expression levels were measured by qPCR and normalized to parental cells cultured in presence of LIF. Gapdh was used as internal control.

(H) Gene expression analysis of parental (OLC2.1), Esrrb overexpressing cells before (Esrrb+) and after (Esrrb-) excision of the transgene. The levels of exogenous Esrrb expression were measured using primers specifically recognising the Esrrb-P2A region of the transgene. Levels are normalized to Esrrb+ cells and Gapdh was used as internal control.

A

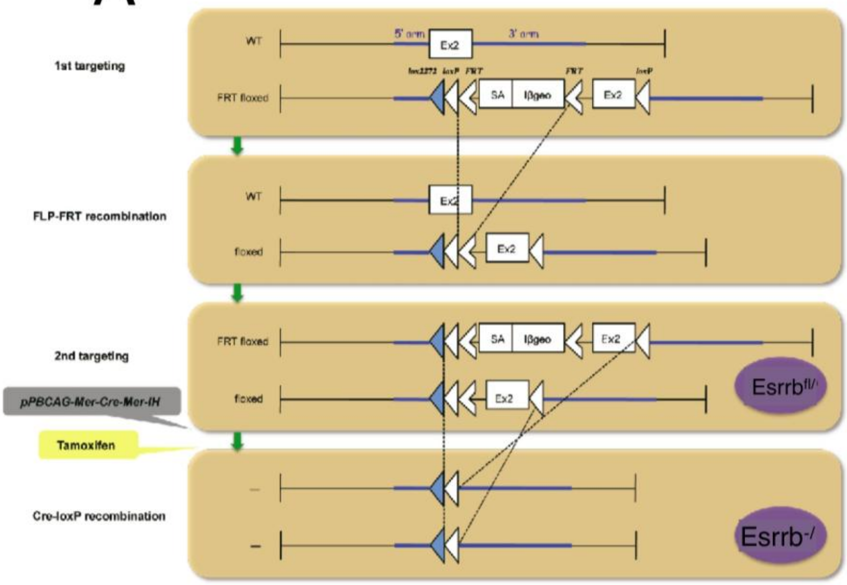

B

Esrrb cKO vector

Ex2	Left Primer (118846-)	ATctcgagagatctAATTCAAAGTTTCTTAGAACTCTG	XhoI, BglII
	Right Primer (-119562)	ACgctagcGGAGACTTCTAGAGCACCTCTGCC	NheI
5' arm	Left Primer (116863-)	ATgcggccgcGGTGGTTAGACAAGGTAGCCATAC	NotI
	Right Primer (-118845)	CTaccggtCTTgtcgacCGTAAACTTCAAATCAGAGACCCT	AgeI, SalI
3' arm	Left Primer (119563-)	CTgtcgacccatggTGATCTGTGTCTCTGGGATCCTAG	SalI, NcoI
	Right Primer (-125909)	GTatcgatCTTGAAACACCTGAAGTTTCCTCT	ClaI

C

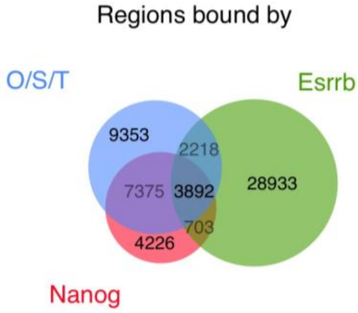

D

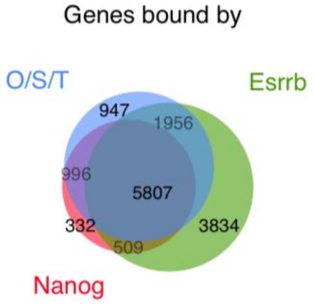

Figure S4. Related to Figure 4

(A) Schematic of strategy used to generate the *Esrrb*$^{fl/fl}$ and *Esrrb*$^{-/-}$ cells.

(B) Primers used for genotyping (see also Methods).

(C) Venn diagram showing the intersection between the genomic regions bound by at least one factor among Oct3/4, Sox2 and Tcf3 (O/S/T in blue), by Nanog (in red) and by Esrrb (in green). In contrast to Figure 4N, all the ChIP-seq peaks have been used for the analysis.

(D) Venn diagram showing the intersection between the predicted target genes bound by at least one factor among Oct3/4, Sox2 and Tcf3 (O/S/T in blue), and the predicted target genes of Nanog (in red) or Esrrb (in green). In contrast to Figure 4O, all the ChIP-seq peaks have been used for the analysis.

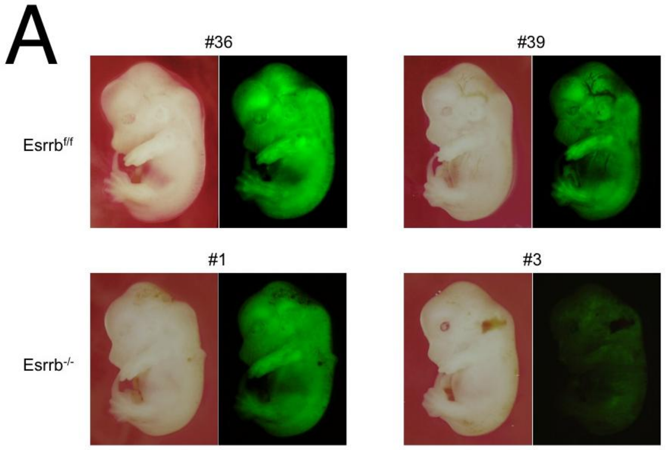

B

	injection	placentation	embryo	chimera
Esrrb$^{f/f}$ #36	10 × 2 × 2 mice	24	10	7
Esrrb$^{f/f}$ #39	10 × 2 × 2 mice	22	6	2
Esrrb$^{-/-}$ #1	10 × 2 × 2 mice	24	14	5
Esrrb$^{-/-}$ #3	10 × 2 × 2 mice	27	12	3 (partial)

Figure S5. Related to Figure 5

(A) Representative pictures of chimeric embryos generated after blastocyst injection of 2 independent Esrrb -/- lines and 2 control fl/fl lines.

(B) Statistics of blastocyst injection experiments performed. All ES cells were cultured in 2i+LIF before blastocyst injection and embryos were analyzed at E12.5 .

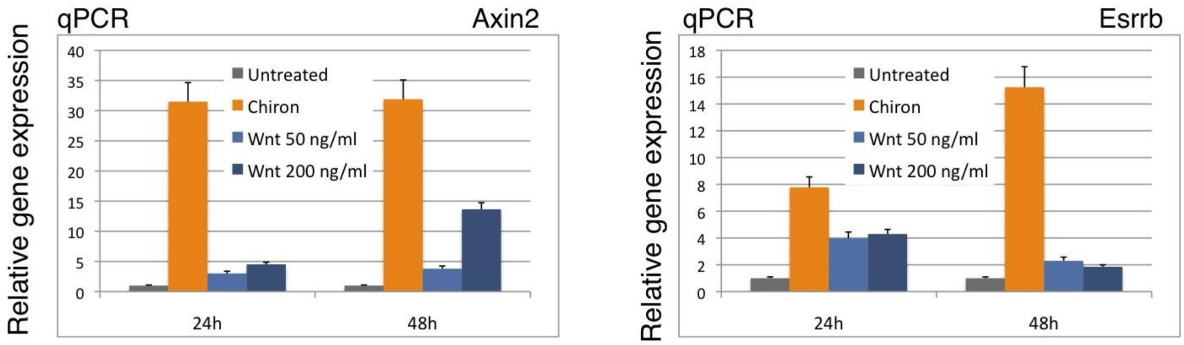

Figure S6. Related to Figure 6

Expression levels of the Wnt target Axin2 (left) and Esrrb (right) after treatment with recombinant Wnt protein or the Gsk3 inhibitor CH for the indicated time. Gene expression levels were measured by qPCR and normalized to cells cultured in LIF/PD. Gapdh was used as internal control. Mean and SD of 2 experiments are shown.

Table S1. ChIP-seq Studies Included in the Compendium

Factor	Antibody	Peaks	Gene Expression Omnibus accession	Publication first author, year (PMID)	Peak source
Aff4	Custom made (see Lin et al. 2010, Mol. Cell)	2659	GSM749810	Lin, 2011 (21764852)	MACS (1e-9)
Brg	Custom made (Clone J1, Khavari et al., 1993)	642	GSM359413	Ho, 2009 (19279218)	MACS (1e-15)
CHD7	Abcam (AB31824)	3428	GSM558674	Schnetz, 2010 (20657823)	Consensus (1)
CTCF	Upstate (07-729)	27843	GSM288351	Chen, 2008 (18555785)	MACS (1e-9)
CTCF	Upstate (07-729)	25121	GSM699165	Handoko, 2011 (21685913)	Consensus (3)
CTCF	Upstate (07-729)	19784	GSM560352	Kagey, 2010 (20720539)	MACS (1e-9)
CTCF (GFP tag)	Custom made (anti-GFP)	33788	GSM634247	Nitzsche, 2011 (21589869)	Originals
Dpy-30	Bethyl Labs (unreleased)	6308	GSM651192	Jiang, 2011 (21335234)	MACS (1e-12)
E2f1	Upstate (05-379)	20557	GSM288349	Chen, 2008 (18555785)	MACS (1e-9)
Ell2	Custom made (see Lin et al. 2010, Mol. Cell)	1949	GSM749809	Lin, 2011 (21764852)	MACS (1e-9)
Eset	Custom made	2577	GSM440256	Yuan, 2009 (19884257)	MACS (1e-9)
Esrrb	Custom made	35808	GSM288355	Chen, 2008 (18555785)	MACS (1e-15)
Ezh2	Active Motif (39103)	4380	GSM327668	Ku, 2008 (18974828)	MACS (1e-9)
Klf4	Custom made	11150	GSM288354	Chen, 2008 (18555785)	MACS (1e-9)
MCAF1	Bethyl Labs (A300-169A)	974	GSM656526	Young lab, 2011 (unpublished)	MACS (1e-9)
Med1	Bethyl Labs (A300-793A)	5447	GSM560347 & GSM560348	Kagey, 2010 (20720539)	MACS (1e-9)*
Med12	Bethyl Labs (A300-774A)	8318	GSM560345	Kagey, 2010 (20720539)	MACS (1e-9)
n-Myc	Custom made	6759	GSM288357	Chen, 2008 (18555785)	MACS (1e-9)
Nanog	Cosmo Bio (REC-RCAB0002PF)	4957	GSM288345	Chen, 2008 (18555785)	MACS (1e-9)
Nanog	Bethyl Labs (A300-397A)	16198	GSM307140 & GSM307141	Marson, 2008 (18692474)	MACS (1e-9)
NelfA	Santa Cruz (sc-23599)	14595	GSM515664	Rahl, 2010 (20434984)	MACS (1e-9)
Nipbl	Bethyl Labs (A301-779A)	11070	GSM560350	Kagey, 2010 (20720539)	MACS (1e-9)
Oct4	Santa Cruz (sc-8628)	2330	GSM566277	Ang, 2011 (21477851)	MACS (1e-12)
Oct4	Santa Cruz (sc-8628)	2768	GSM288346	Chen, 2008 (18555785)	MACS (1e-9)
Oct4	Santa Cruz (sc-8628)	15701	GSM307137	Marson, 2008 (18692474)	MACS (1e-9)
p300	Santa Cruz (sc-585)	14355	GSM594600	Creyghton, 2010 (21106759)	MACS (1e-12)
p300	Santa Cruz (sc-585)	628	GSM558675	Schnetz, 2010 (20657823)	Consensus (1)
p300	Santa Cruz (sc-585)	2575	GSM699164	Handoko, 2011 (21685913)	Consensus (2)
Prdm14 (FLAG and HA tag)	Sigma (A2220 & A2095)	26559	GSM623989	Ma, 2011 (21183938)	MACS (1e-15)
Rad21 (GFP tag)	Custom made (anti-GFP)	15311	GSM591469	Nitzsche, 2011 (21589869)	Originals
Rbbp5	Custom made (Allis Lab)	5326	GSM566278	Ang, 2011 (21477851)	MACS (1e-12)
REST	Upstate (07-579)	1547	GSM656525	Young lab, 2011 (unpublished)	MACS (1e-9)
Ring1b	Custom made	5167	GSM656523	Young, 2011 (unpublished)	MACS (1e-9)
Sin3A	Abcam (AB3479)	8047	GSM611196	Williams, 2011 (21490601)	MACS (1e-15)
Sin3A	Santa Cruz (sc-994X)	3423	GSM611197	Williams, 2011 (21490601)	MACS (1e-15)

Smc1	Bethyl Labs (A300-055A)	9777	GSM560341 & GSM560342	Kagey, 2010 (20720539)	MACS (1e-9)*
Smc3	Abcam (AB9263)	9715	GSM560343 & GSM560344	Kagey, 2010 (20720539)	MACS (1e-9)*
Sox2	Santa Cruz (sc-17320)	3683	GSM288347	Chen, 2008 (18555785)	MACS (1e-9)
Sox2	R&D Systems (AF2018)	16721	GSM307138 & GSM307139	Marson, 2008 (18692474)	MACS (1e-9)
Spt5	Custom made (see Wada et al., 1998)	3232	GSM515665	Marson, 2010 (20434984)	MACS (1e-9)
Stat3	Upstate (sc-482)	1353	GSM288353	Chen, 2008 (18555785)	MACS (1e-12)
Stat3_BrgKO	Santa Cruz (sc-482 X)	185	GSM686674	Ho, 2011 (21785422)	Consensus (4)
Stat3_BrgWT	Santa Cruz (sc-482 X)	992	GSM686673	Ho, 2011 (21785422)	Consensus (4)
Suz12	Abcam (AB12073)	3900	GSM288360	Chen, 2008 (18555785)	MACS (1e-9)
Suz12	Abcam (AB12073)	4793	GSM307144 & GSM307145	Marson, 2008 (18692474)	MACS (1e-9)
TBP	Abcam (AB818)	3629	GSM555160 & GSM555162	Young lab, 2010 (unpublished)	MACS (1e-12)*
Tcf3	Santa Cruz (sc-8635)	5755	GSM307142 & GSM307143	Marson, 2008 (18692474)	MACS (1e-9)
Tcfcp2l1	Custom made	20666	GSM288350	Chen, 2008 (18555785)	MACS (1e-15)
Tet1	Custom made	2969	GSM706672	Xu, 2011 (21514197)	MACS (1e-12)
Tet1	Custom made	24033	GSM659799	Wu, 2011 (21451524)	MACS (1e-9)
Tet1-C	Custom made	26912	GSM611192	Williams, 2011 (21490601)	MACS (1e-15)
Tet1-N	Custom made	14644	GSM611194	Williams, 2011 (21490601)	MACS (1e-15)
Wdr5	Bethyl Labs (A302-429A)	1327	GSM566279	Ang, 2011 (21477851)	MACS (1e-12)
Wdr5 (FLAG tag)	Sigma (F3165)	4331	GSM566280	Ang, 2011 (21477851)	MACS (1e-12)
Zfx	Custom made	14187	GSM288352	Chen, 2008 (18555785)	MACS (1e-9)

Notes: The table summarizes the ChIP-Seq studies currently included which totals 55 independent experiments covering 38 different factors; read density profiles, peak files and lists of candidate target genes for all 55 factors are available for download at (http://bioinformatics.cscr.cam.ac.uk/ES_Cell_ChIP-seq_compendium.html) **(1)** Originals and MACS (1e-9); **(2)** MACS (1e-9) and PeakSeq (0.05); **(3)** MACS (1e-15) and PeakSeq (0.01); **(4)** MACS (1e-15) and PeakSeq (0.05); * Intersect of replicates.

Table S2. qPCR primers sequences used for qPCR

UPL PRIMERS

Gene Name	Forward	Reverse	UPL probe
Fgf5	aaaacctggtgcaccctaga	catcacattcccgaattaagc	29
Klf2	ctaaaggcgcatctgcgta	tagtggcgggtaagctcgt	48
Klf4	cgggaagggagaagacact	gagttcctcacgccaacg	62
Klf5	ccggagacgatctgaaacac	cagatacttctccatttcacatcttg	17
Nanog	ttcttgcttacaagggtctgc	agaggaagggcgaggaga	110
Nr0B1	cgtgctctttaacccagacc	ccggatgtgctcagtaagg	**3**
Pou5f1	gttggagaaggtggaaccaa	ctccttctgcagggctttc	95
Rex1	tcttctctcaatagagtgagtgtgc	gctttcttctgtgtgcagga	71
Sox2	tccaaaaactaatcacaacaatcg	gaagtgcaattgggatgaaaa	63
Tcfcp2l1	gaccatttcaagcactgaccta	caaagcatggaagaacatgg	4

SYBR Green primers (used in figures 4F, K and L and S4)

Gene name	FORWARD	REVERSE
Esrrb-P2A	CCTGCTGAGGCAGACAGCCG	GGTTCTCCTCCACGTCGCCG
Esrrb (endogenous or Tg)	CTCGCCAACTCAGATTCGAT	AGAAGTGTTGCACGGCTTTG
Esrrb (Ex2)	CATGGAGGACTCCGCCATC	CTTGAATGGTTCTCTTGAAG
Oct3/4	CACGAGTGGAAAGCAACTCA	AGATGGTGGTCTGGCTGAAC
Sox2	AAGGGTTCTTGCTGGGTTTT	AGACCACGAAAACGGTCTTG
Nanog	ACCTGAGCTATAAGCAGGTTAAGAC	GTGCTGAGCCCTTCTGAATCAGAC
Klf4	CCAGCAAGTCAGCTTGTGAA	GGGCATGTTCAAGTTGGATT
Klf5	GCCAGTTAATTCGCCAACTC	CCCGTATGAGTCCTCAGGTG
Tbx3	ATCTGCCAGTGCACTTTGTTAGATG	TGTTCTTCAGCCCCGACTTCCATAC
Nr5a2	ATGGGAAGGAAGGGACAATC	ATACAAACTCCCGCTGATCG
Zfp42	GACAGAGGCACTGGGGATAC	CGTATGCAAAAGTCCCCATC

Table S3. siRNAs Used in This Study

Name	Cat. From Qiagen
siControl	1027280
si Esrrb A	SI02672110
si Esrrb B	SI02739569
si Klf2 A	SI01083523
si Klf2 B	SI01083544
si Nanog A	SI04460869
si Nanog B	SI01323357
si Nr0B1 A	SI02666125
si Nr0B1 B	SI02685809
si Tcfcp2l1 A	SI01444296
si Tcfcp2l1 B	SI04401558
siGFP	custom (target sequence GCAAGCTGACCTGAAGTTCA)

Table 4. qPCR Primers Sequences Used for Tcf3 ChIP

Region1 F	aaaggtaggcccgatttcac
Region1 R	aaaatagcccagagcttccag
Region2 F	atcagttcagggacagttgagag
Region2 R	cctaaagctcagagcacatgg
Region3 F	ccaactggagtaaagatgtcca
Region3 R	cattcactgcaggcacctc
Region4 F	tcattggcaaagaggacaaa
Region4 R	cggatctgtggaattcgtg
Region5 F	gaagtggttcttgccggtta
Region5 R	gtaactaacatcctctccctgagc

Supplemental Experimental Procedures

Chromatin Immunoprecipitation

Embryonic stem cells ($3x10^6$ for each sample) were fixed for 10 min in 1% formaldehyde, collected in cold PBS and incubated for 20 min on ice in swelling buffer (5 mM HEPES at pH 8.25, 85 mM KCl and 0.5% NP-40). Nuclei were pelleted in a microfuge and resuspended in lysis buffer (10 mM Tris at pH 8.0, 1 mM EDTA, 0.5 mM EGTA and 1% SDS). Lysates were sonicated to obtain an average DNA fragment size of ~500 base pairs. Lysates were diluted 1:10 in chromatin immunoprecipitation dilution buffer (50 mM Tris-HCl at pH 8.0, 167 mM NaCl, 1.1% Triton X-100 and 0.11% Na deoxycholate), pre-cleared for 2 h at 4 ◦ C with protein-G Sepharose beads (Amersham) and incubated overnight at 4 ◦ C with 2µg Tcf3 antibody (Santa Cruz, sc8635) or an isotype IgG antibody as a control (Santa Cruz, sc2028). Lysates were then incubated for 30 min at 4 ◦ C with blocked protein-G Sepharose beads, beads were washed twice each in radioimmunoprecipitation assay buffer (RIPA; 100 mM Tris-HCl at pH 8.0, 300 mM NaCl, 2 mM EDTA at pH 8.0, 2% Triton X-100, 0.2% SDS and 0.2% Na deoxycholate), RIPA/150 mM NaCl and Tris-EDTA buffer. Chromatin was eluted for 30 min at room temperature in elution buffer (100 mM Tris-HCl at pH 8.0, 300 mM NaCl, 5 mM EDTA at pH 8.0 and 0.5% SDS), Samples were incubated overnight at 65°C to reverse the cross-linking and purified using QIAquick PCR Cleanup kit (Qiagen). Chromatin was analysed by SYBR green real-time PCR (see primers in Supplementary Table S4). Enrichment was calculated relative to the IgG ChIP.

Immunostaining

Cells were fixed for 10 minutes in 4% PFA at Room Temperature (RT), permeabilized for 5 minutes in PBS+0.2% TritonX at RT, and blocked for 30 minutes in PBS+3% donkey serum ar RT. Cells were incubated overnight at 4◦ C with an anti-Esrrb monoclonal antibody (Perseus, pp-H6705-00, 1:500 in PBS). After washing in PBS the cells were incubated with a donkey anti-Mouse IgG Alexa Fluor-555-conjugated antibody (A31570 from Invitrogen, 1:500 in PBS), for 30 minutes at RT. After DAPI staining, more than 10 images for each sample (>3000 cells) were

acquired using a Zeiss AxioObserver D1 microscope. DAPI labelled cells were indentified using Volocity software (Perkin Elmer) and intensity of 455-labelled Esrrb measured. Intensity measurements were expressed as a frequency distribution using Excel (Microsoft) to categorize negative, intermediate and positive cells.

Immunoblotting

Immunoblotting was perfomed as previously described in Yang et al., 2010. Antibodies used: mouse monoclonal anti-Esrrb ((Perseus Proteomics, pp-H6705-00, 1:1000 dilution in 5% milk); mouse monoclonal anti-GAPDH (Sigma-Aldrich, G8795, 1:1000 in 5% milk).
